# Prrx1b restricts fibrosis and promotes Nrg1-dependent cardiomyocyte proliferation during zebrafish heart regeneration

**DOI:** 10.1242/dev.198937

**Published:** 2021-10-04

**Authors:** Dennis E. M. de Bakker, Mara Bouwman, Esther Dronkers, Filipa C. Simões, Paul R. Riley, Marie-José Goumans, Anke M. Smits, Jeroen Bakkers

**Affiliations:** 1 Hubrecht Institute-KNAW and University Medical Center Utrecht, 3584CT Utrecht, The Netherlands; 2 Department of Cell and Chemical Biology, Leiden University Medical Centre, 2333ZC Leiden, The Netherlands; 3 Department of Physiology, Anatomy and Genetics, University of Oxford, Oxford OX1 3PT, UK; 4 Department of Pediatric Cardiology, University Medical Centre Utrecht, 3584CX Utrecht, The Netherlands

**Keywords:** Prrx1, Neuregulin, Zebrafish, Heart regeneration, Fibrosis, Fibroblasts

## Abstract

Fibroblasts are activated to repair the heart following injury. Fibroblast activation in the mammalian heart leads to a permanent fibrotic scar that impairs cardiac function. In other organisms, such as zebrafish, cardiac injury is followed by transient fibrosis and scar-free regeneration. The mechanisms that drive scarring versus scar-free regeneration are not well understood. Here, we show that the homeobox-containing transcription factor Prrx1b is required for scar-free regeneration of the zebrafish heart as the loss of Prrx1b results in excessive fibrosis and impaired cardiomyocyte proliferation. Through lineage tracing and single-cell RNA sequencing, we find that Prrx1b is activated in epicardial-derived cells where it restricts TGFβ ligand expression and collagen production. Furthermore, through combined *in vitro* experiments in human fetal epicardial-derived cells and *in vivo* rescue experiments in zebrafish, we conclude that Prrx1 stimulates Nrg1 expression and promotes cardiomyocyte proliferation. Collectively, these results indicate that Prrx1 is a key transcription factor that balances fibrosis and regeneration in the injured zebrafish heart.

This article has an associated ‘The people behind the papers’ interview.

## Abstract (precis)

**Summary:** Epicardial-expressed Prrx1 ensures a balance between the fibrotic response and myocardial regeneration post-injury in zebrafish.

## INTRODUCTION

The regenerative capacity of damaged organs and body parts differs widely within the animal kingdom, which is particularly true for the heart (Nguyen et al., 2021; Poss, 2010). Indeed, whereas zebrafish display robust regeneration after heart injury, the wound response in mammalian hearts does not include replenishment of the lost myocardium. Instead, the affected myocardium is permanently lost and replaced by a fibrotic scar, which does not contribute to cardiac contraction resulting in reduced cardiac output.

The fibrotic scar is formed by cardiac fibroblasts that become activated to produce large amounts of extracellular matrix (ECM) components, such as collagens. Cardiac fibroblasts mainly originate from the embryonic epicardium, which consists of a heterogeneous population of epithelial cells that cover the heart (Acharya et al., 2012; Cao et al., 2016; Gittenberger-de Groot et al., 1998; Hortells et al., 2020; Travers et al., 2016; Weinberger et al., 2020). During embryonic heart development, a subset of epicardial cells undergoes an epithelial-to-mesenchymal transition (EMT) and migrates into the cardiac wall to give rise to a variety of cell types, which are commonly referred to as epicardial-derived cells (EPDCs) and include predominantly cardiac fibroblasts and vascular support cells (Cao and Poss, 2018).

In contrast to mammals, adult zebrafish can fully regenerate their heart after resection or cryoinjury of 20-30% of the ventricle (Chablais et al., 2011; González-Rosa et al., 2011; Poss et al., 2002; Schnabel et al., 2011), as a result of reactivation of proliferation in border zone cardiomyocytes (Jopling et al., 2010; Kikuchi et al., 2010; Wu et al., 2015). One of the first responses upon injury is the activation of a developmental gene expression programme in the epicardium, at 1-2 days after the injury (Lepilina et al., 2006). This response becomes confined to the injury area as the epicardium is regenerated, which completely surrounds the injury area between 3 and 7 days post-injury (dpi) (Kikuchi et al., 2011; Lepilina et al., 2006). The epicardium is essential for the regeneration process as ablating *tcf21*-expressing epicardial cells from the injured zebrafish heart impairs cardiomyocyte proliferation and regeneration (Wang et al., 2015). Similar to observations in the mammalian heart, lineage tracing of *wt1b-* and *tcf21*-expressing cells in zebrafish revealed that the embryonic epicardium gives rise to EPDCs, such as cardiac fibroblasts and vascular support cells (González-Rosa et al., 2012; Kikuchi et al., 2011; Sánchez-iranzo et al., 2018). Upon injury, EPDCs secrete signals guiding regeneration such as TGFβ ligands, platelet derived growth factor, cytokines, such as Cxcl12, and mitogenic factors, such as Nrg1 (Chablais and Jaźwińska, 2012; Gemberling et al., 2015; Itou et al., 2012; Kim et al., 2010). In addition, EPDCs also contribute to fibrosis by producing ECM components, such as collagen (Sánchez-iranzo et al., 2018). Fibrosis in the zebrafish heart is transient and regresses as regeneration proceeds, which coincides with the inactivation of cardiac fibroblasts, ultimately resulting in a scar-free heart (Chablais et al., 2011; Sánchez-iranzo et al., 2018). Although EPDCs play important roles during fibrosis and pro-regenerative signalling, the molecular mechanism regulating these processes remains largely unknown.

The paired related homeobox 1 (Prrx1) gene encodes a transcription factor and its expression correlates with scar-free wound healing and limb regeneration (Satoh et al., 2010; Stelnicki et al., 1998; Yokoyama et al., 2011). Although its function has been studied during embryonic development, its role during wound healing or regeneration remains largely unknown. Here, we find that Prrx1b expression is induced in the epicardium and EPDCs during zebrafish heart regeneration and we show that Prrx1b is required for scar-free regeneration. By single-cell RNA sequencing of EPDCs, we identified that loss of *prrx1b* results in an excess of pro-fibrotic fibroblasts and fibrosis. Furthermore, we find that Prrx1b is necessary for the induction of *nrg1* signalling, which we confirmed in an *in vitro* system of human fetal EPDCs. Altogether, our data indicate that Prrx1b regulates zebrafish heart regeneration by maintaining the balance between the fibrotic and regenerative responses after heart injury.

## RESULTS

### *prrx1b* is required for zebrafish cardiomyocyte proliferation and heart regeneration

**Fig. 1.**
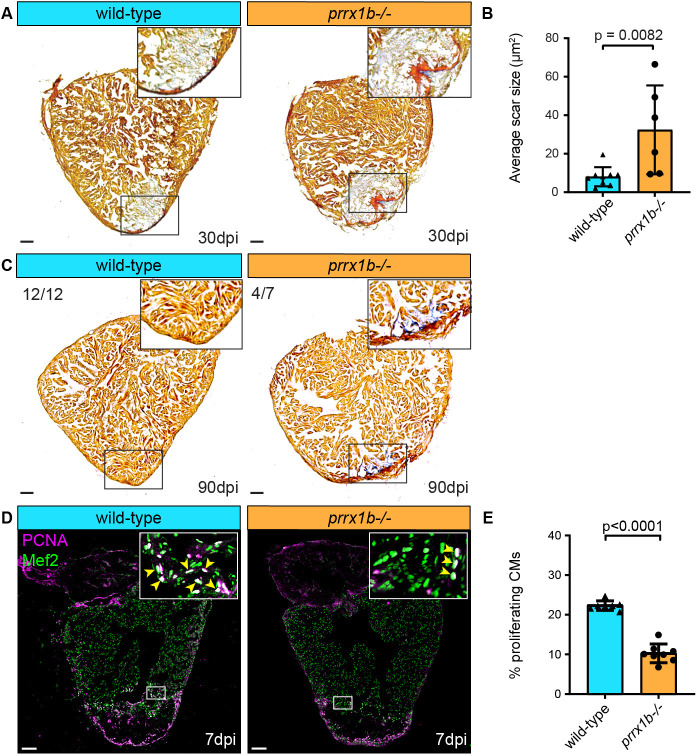
**Heart regeneration and border zone cardiomyocyte proliferation is impaired in *prrx1b*^−/−^ zebrafish.** (A) AFOG staining on 30 dpi wild-type and *prrx1b*^−/−^ heart sections showing fibrin in red, collagen in blue and remaining muscle tissue in orange. Scale bars: 100 μm. (B) Quantification of the remaining scar size at 30 dpi in *prrx1b*^−/−^ hearts (*n*=6) and wild-type sibling hearts (*n*=9) (mean±s.d., *P*=0.0082, unpaired *t*-test). (C) AFOG staining on sections of 90 dpi hearts. Scars were completely resolved in wild-type hearts (*n*=12), whereas incomplete scar resolution was observed in *prrx1b*^−/−^ four out of seven hearts. Scale bars: 100 μm. (D) Immunofluorescence staining on 7 dpi wild-type and *prrx1b*^−/−^ heart sections using an anti-Mef2 antibody as a marker for cardiomyocyte nuclei, and an anti-PCNA antibody as a nuclear proliferation marker. Insets show higher magnifications of the boxed areas. Arrowheads indicate proliferating cardiomyocytes. Scale bars: 100 μm (main panels); 10 μm (insets). (E) Quantification of the percentage of proliferating (PCNA^+^) border zone cardiomyocytes in *prrx1b*^−/−^ hearts (*n*=8) and wild-type sibling hearts (*n*=7) (mean±s.d., *P*<0.0001, unpaired *t*-test).

In order to address a potential role for Prrx1 in heart regeneration, we utilized *prrx1a*^−/−^ and *prrx1b*^−/−^ adult fish, which harbour nonsense mutations upstream of the conserved DNA-binding domain (Barske et al., 2016). Both *prrx1a*^−/−^ and *prrx1b*^−/−^ display no developmental defects owing to redundant gene functions and only *prrx1a*^−/−^*;prrx1b*^−/−^ embryos display craniofacial defects (Barske et al., 2016). We subjected adult *prrx1a*^−/−^ and *prrx1b*^−/−^ fish to cardiac cryoinjury and analysed scar sizes at 30 dpi. Although we observed comparable scar sizes in wild-type and *prrx1a*^−/−^ hearts, scar sizes were significantly larger in *prrx1b*^−/−^ hearts compared with their control siblings (Fig. 1A,B, Fig. S1A). This difference in scar size was still apparent at 90 dpi, when wild-type hearts had completely resolved their scars (12/12) whereas the majority of *prrx1b*^−/−^ hearts (4/7) had not (Fig. 1C). Because myocardial regeneration is achieved through proliferation of the surviving cardiomyocytes at the injury border zone, we investigated cardiomyocyte proliferation in the *prrx1a*^−/−^ and *prrx1b*^−/−^ hearts. Indeed, *prrx1b*^−/−^ hearts showed a significant reduction in border zone cardiomyocyte proliferation at 7 dpi whereas no significant differences were observed in *prrx1a*^−/−^ hearts (Fig. 1D,E, Fig. S1B,C). From these results, we conclude that *prrx1b*, but not *prrx1a*, is required for zebrafish cardiomyocyte proliferation and heart regeneration.

### Prrx1 is expressed in epicardial and epicardial-derived cells

To address how Prrx1b could function during zebrafish heart regeneration, we investigated the spatial distribution of Prrx1b expression. By mRNA *in situ* hybridization (ISH) we observed only a very weak signal for *prrx1b* on sections of 7 dpi hearts with some signal in the (sub)epicardium close to the border zone and some signal in the injury area (Fig. S2A). As these weak signals were difficult to interpret, we instead used an antibody raised against the N terminus of the axolotl Prrx1 protein that recognizes zebrafish Prrx1 (Gerber et al., 2018; Ocaña et al., 2017). Prrx1 protein was mainly detected in (sub)epicardial cells covering the injury area and some sparse cells within the injury (Fig. S2B). Importantly, we could validate that the Prrx1 antibody recognizes the zebrafish Prrx1b protein as injured *prrx1b*^−/−^ hearts showed a near lack of the signal (Fig. S2B,C). In addition, Prrx1 protein was nearly undetectable in uninjured zebrafish hearts (Fig. S2D), together indicating that Prrx1 protein expression is induced upon heart injury and at 7 dpi localizes in cells covering and within the injury area.

**Fig. 2.**
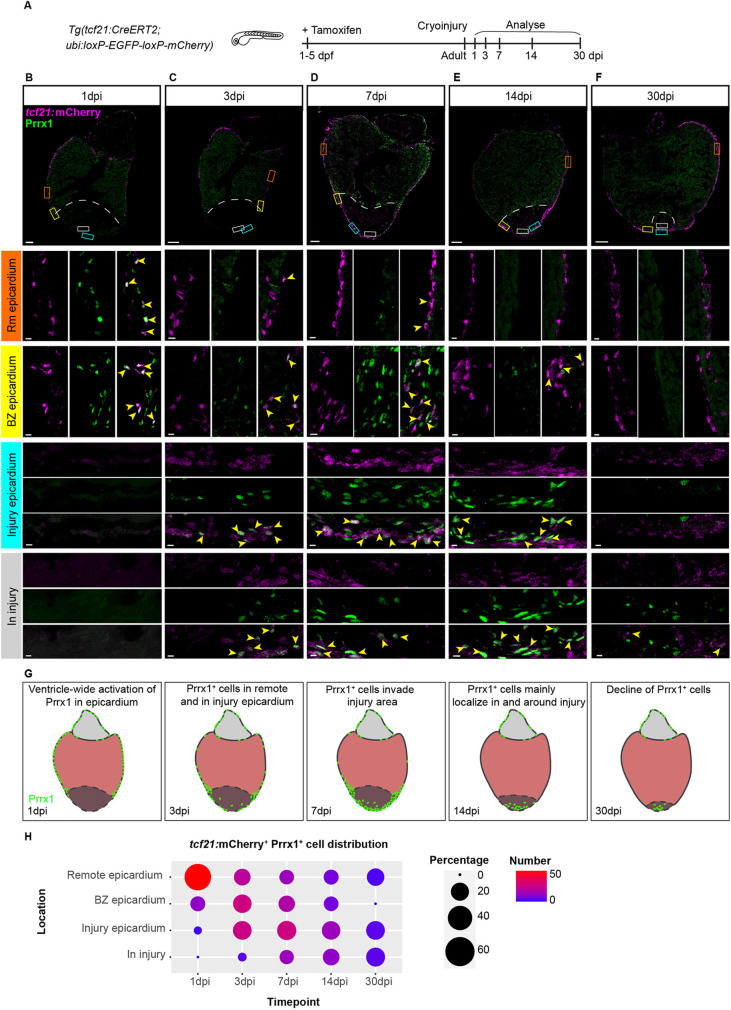
**Prrx1 is expressed in the epicardium/EPDCs and follows epicardial dynamics post-injury.** (A) Schematic illustrating the experimental procedures. (B-F) Immunofluorescence staining on 1, 3, 7, 14 and 30 dpi *tcf21:*mCherry^+^ wild-type heart sections staining Prrx1 (green) and mCherry (magenta). Areas in the coloured boxes are shown at higher magnification below. Arrowheads indicate *tcf21*:mCherry^+^/Prrx1^+^ cells. Scale bars: 100 μm (low-magnification images); 10 μm (high-magnification images). BZ epicardium, border zone epicardium; Rm epicardium, remote epicardium. Six hearts analysed per condition. Dashed line indicates the border between myocardium and injury area. (G) Schematic of Prrx1 dynamics upon injury. Prrx1^+^ cells are in green. Dark colour at the apex represents the injury area. (H) Quantification of the distribution of *tcf21:*mCherry^+^/Prrx1^+^ cells per time point. Size of the dots represents the percentage of *tcf21:*mCherry^+^/Prrx1^+^ cells and absolute count number is visualized by a colour gradient.

As this localization of Prrx1 suggested a possible expression in EPDCs, we used the *Tg(tcf21:CreERT2)* line, which marks a subset of EPDCs when crossed with the ubiquitous reporter *Tg(ubi:loxP-EGFP-loxP-mCherry)* and recombined during embryonic heart development (Kikuchi et al., 2011). Hearts from embryonic recombined *Tg(tcf21:CreERT2; ubi:loxP-EGFP-loxP-mCherry)* fish were cryo-injured, extracted at different time points and analysed for Prrx1 and mCherry expression (Fig. 2A-F). At 1 dpi, we observed a strong expression of Prrx1 in the entire intact epicardium (Fig. 2B), coinciding with the previously reported ventricle-wide injury response of the epicardium (Lepilina et al., 2006). At 3 dpi and 7 dpi, we started to observe double-labelled mCherry^+^ and Prrx1^+^ cells around and in the injury area whereas Prrx1 expression became less dense in the remote ventricle (Fig. 2C,D,H). Interestingly, from 1 to 7 dpi we observed an accumulation of mCherry/Prrx1^+^ cells in the regions where the (sub)epicardium is close to the border between injured and uninjured myocardium (the so-called border zone, BZ), which we refer to as the BZ epicardium (Fig. 2B-D,H). At 14 dpi, we observed the majority of Prrx1 expression in and around the injury area (Fig. 2E,H). At 30 dpi, only a few mCherry/Prrx1^+^ cells were detected, of which the majority was located at the apex and inside the remaining injury area (Fig. 2F,H). Importantly, although at all time points Prrx1 was mostly found in mCherry^+^ cells, not all mCherry^+^ cells were Prrx1^+^ and vice versa, confirming the previously reported heterogeneity of the epicardium and EPDCs (González-Rosa et al., 2012). To address whether other epicardial subpopulations express Prrx1, we analysed Prrx1 expression in *Tg(tbx18:myr-eGFP)* and *Tg(wt1b:H2B-Dendra2)* hearts. Indeed, we observed that Prrx1 is co-expressed with *tbx18* and *wt1b* (Fig. S3). Taken together, these results indicate that upon cardiac injury Prrx1 expression is induced in *tcf21^+^*, *tbx18^+^* and *wt1^+^* epicardial subpopulations. Prrx1 expression starts in the epicardium covering the remote myocardium, after which it becomes more restricted to the injury epicardium followed by expression in EPDCs localized in the injury area at later stages.

### Proliferation and invasion of epicardial cells is unaffected in *prrx1b^−/−^* hearts

Because cardiac injury induces the proliferation of epicardial cells (Lepilina et al., 2006) and Prrx1b is expressed in this cell type, we decided to investigate whether *prrx1b* plays a role in epicardial cell proliferation. To do so, we used the *Tg(tcf21:CreERT2; ubi:loxP-EGFP-loxP-mCherry)* line to identify epicardial cells in wild-type and *prrx1b*^−/−^ hearts and used PCNA expression to identify proliferating cells (Fig. S4A,B). We observed that the number of PCNA-expressing epicardial cells was highest at 1 and 3 dpi, after which their number dropped to significantly lower amounts at 30 dpi, which is in line with previous reports (Lepilina et al., 2006). We found no indication of an epicardial proliferation defect in *prrx1b^−/−^* hearts, as no significant differences were observed in the number of PCNA-expressing epicardial cells at any of the examined time points (Fig. S4B).

Furthermore, we wondered whether *prrx1b* could play a role during the invasion of EPDCs into the injury area. To quantify this, we determined the contribution of *tcf21*:mCherry^+^ surface area found within the injury area as a proportion of the total *tcf21*:mCherry^+^ surface area found in and around the injury area (Fig. S4C). We used this percentage as a measure of invasion efficiency of the EPDCs. At all time points, except for 3 dpi, there was no significant difference in the percentage of mCherry^+^ cells inside the injury between the wild-type and *prrx1b^−/−^* hearts. At 3 dpi, the percentage of mCherry^+^ cells invading the injury area was significantly lower in the *prrx1b^−/−^* hearts, but this was mitigated from 7 dpi onwards.

Taken together, we did not find evidence suggesting a profound role for Prrx1 in epicardial proliferation or EMT. Although the incomplete labelling of *tcf21*^+^ cells resulted in substantial variation in the collected data, potentially masking a subtle difference between wild-type and *prrx1b^−/−^* hearts, we conclude that Prrx1 is largely dispensable for proliferation and invasion into the injury area of epicardial and epicardial-derived cells.

### Characterization of EPDCs in the regenerating heart by single-cell sequencing

**Fig. 3.**
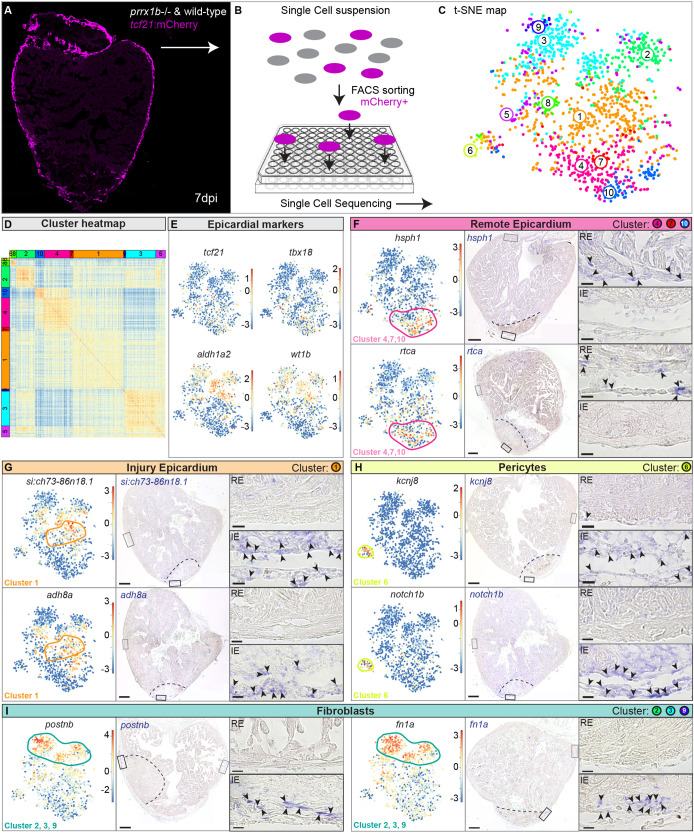
**Single-cell sequencing identifies epicardial-derived cell populations in the injured zebrafish heart.** (A,B) Workflow of the isolation (A) and sorting (B) of *tcf21:*mCherry^+^ cells in wild-type and *prrx1b*^−/−^ hearts at 7 dpi. (C,D) tSNE plotting of the data results in ten transcriptionally distinct clusters (C), as also indicated by the heatmap (D). (E) tSNE maps visualizing log2-transformed read-counts for *tcf21*, *tbx18*, *aldh1a2* and *wt1b*. (F-I) Characterization of the different cell clusters. Left: Panels show tSNE maps visualizing log2-transformed read-counts for genes with high expression in the indicated cluster (circled). Middle: *In situ* hybridization for the cluster-enriched genes in wild-type hearts at 7 dpi. Dashed line indicates injury border. Scale bars: 100 μm. Right: Magnifications of the boxed regions in remote (RE) and injury epicardium (IE) with arrowheads pointing to cells with high expression. Scale bars:10 μm. Three hearts analysed per condition. Gene lists are provided in Table S1.

Next, we wanted to identify which processes are regulated by Prrx1b in EPDCs that could explain the impaired regenerative response observed in the *prrx1b*^−/−^ hearts. Given that the epicardium and EPDCs are formed by heterogenous cell populations, we decided to take a single-cell RNA sequencing (scRNAseq) approach to characterize these cells within the context of heart regeneration. First, we isolated ventricles from recombined *Tg(tcf21:CreERT2; ubi:loxP-EGFP-loxP-mCherry)* wild-type sibling or *prrx1b^−/−^* fish at 7 dpi and identified the mCherry^+^ cells by fluorescence-activated cell sorting (FACS) (Fig. S5A-C). Next, we performed scRNAseq using the SORT-seq (Sorting and Robot-assisted Transcriptome Sequencing) platform on the isolated single cells (Muraro et al., 2016) (Fig. 3A,B). We analysed over 1400 cells with >1000 reads per cell using the RaceID3 algorithm, which resulted in the identification of ten cell clusters grouped based on their transcriptomic features (Fig. 3C,D, Table S1).

To confirm that the sorting strategy worked, we plotted the expression of the epicardial markers *tcf21*, *tbx18*, *aldh1a2* and *wt1b* and observed that most cells express at least one of these albeit in different patterns (Fig. 3E). These differences in *tcf21*, *tbx18*, *aldh1a2* and *wt1b* expression confirm the previously observed heterogeneity of epicardial cells and EPDCs (Cao et al., 2016; Weinberger et al., 2020). Unfortunately, *prrx1b* expression was too low to correlate it to any of the clusters (Fig. S6). To identify different cell types, we selected marker genes for known cell types and analysed their expression in the various clusters. The cells from clusters 4, 7 and 10 expressed *tcf21* and *tbx18* but lacked expression of *aldh1a2* and *wt1b*, suggesting that these represent epicardial cells covering the remote myocardium. Indeed, ISH for additional genes with high expression in these clusters labelled epicardial cells covering the uninjured part of the heart (Fig. 3F). By contrast, *wt1b* and *aldh1a2* were expressed in most of the remaining clusters, with highest expression in cluster 1. ISH for additional genes marking cluster 1 revealed that their expression was mostly located in the epicardial region covering the injury area but not the remote myocardium (Fig. 3G).

As the epicardium gives rise to fibroblasts and pericytes, we plotted known marker genes for these cell types. Pericytes express genes such as the potassium channel *kcnj8* and the Notch receptor *notch1b* (Guichet et al., 2015; Vanlandewijck et al., 2018). The expression of these genes was most abundant in cluster 6 and Gene Ontology analysis revealed that cluster 6 has an enrichment in genes linked to smooth muscle contraction and cell junction organization. These findings suggest that cells in cluster 6 represent pericytes (Fig. 3H). Periostin (*postnb*) and fibronectin (*fn1a*) are expressed in fibroblasts and both genes showed highest expression in clusters 2, 3 and 9 (Fig. 3I). In addition, we observed that these clusters are enriched for various other genes reported to be expressed in fibroblasts in the context of tissue injury (e.g. *dkk3b*, *fstl1b*, *ptx3a*) (Table S2), suggesting that these clusters are formed by injury-responsive cardiac fibroblasts (Sánchez-iranzo et al., 2018). Gene Ontology analyses with genes enriched in clusters 2, 3 and 9 indeed indicated processes such as ‘extracellular matrix organization’, ‘dissolution of fibrin clot’ and ‘collagen biosynthesis and modifying enzymes’ (Table S3).

From these results, we conclude that the scRNAseq data represent different populations of epicardial and epicardial-derived cells from the regenerating heart. Based on our analysis, we divided the cells into four general groups: remote epicardium (clusters 4, 7 and 10), injury epicardium (cluster 1), epicardial-derived fibroblasts (clusters 2, 3 and 9) and epicardial-derived pericytes (cluster 6).

### Prrx1 restricts fibrosis

**Fig. 4.**
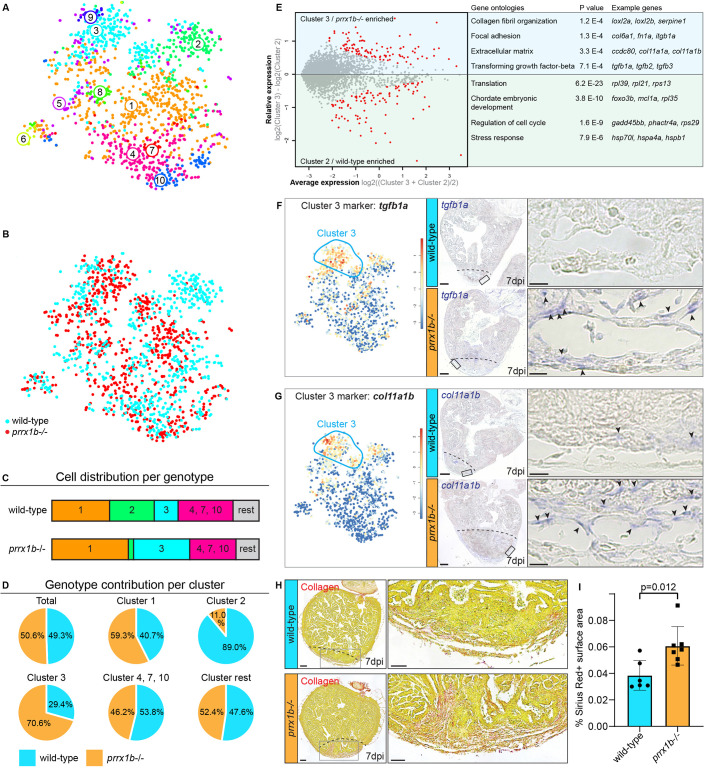
***prrx1b^−/−^* hearts contain excessive amounts of pro-fibrotic fibroblasts.** (A) tSNE map of the single-cell sequencing data as shown in Fig. 3C, indicating ten transcriptionally distinct cell populations. (B) tSNE map showing the contribution of wild-type cells (cyan) and *prrx1b*^−/−^ cells (red). (C) Stacked bar graph showing the relative cell contribution to major clusters in wild-type and *prrx1b*^−/−^ hearts. (D) Pie charts showing the contribution of wild-type and *prrx1b*^−/−^ cells per cluster. (E) Differential gene expression analysis using the DESeq algorithm between fibroblast clusters 2 and 3. Enriched genes were selected for either cluster 2 or 3 with a *P*-value cut-off of <0.05 (red). Gene Ontology analysis was performed using the online tool DAVID. Gene and full Gene Ontology lists are provided in Tables S2 and S3. (F,G) Characterization of cluster 3. Left: tSNE maps visualizing log2-transformed read-counts for genes with high expression in the indicated cluster (circled). Middle: *In situ* hybridization for the cluster 3-enriched genes in wild-type and *prrx1b*^−/−^ hearts at 7 dpi. Dashed line indicates injury border. Scale bars: 100 μm. Right: Magnifications of the boxed regions in the injury area with arrowheads pointing to cells with high expression. Scale bars: 25 μm. Three hearts analysed per condition. (H) Sirius Red staining showing collagen in red on sections of wild-type and *prrx1b*^−/−^ hearts at 7 dpi. Right-hand panels show magnifications of the boxed regions in the sub-epicardial layer and further inside the injury area. Scale bars:100 μm (left); 50 μm (right). (I) Quantification of Sirius Red (collagen) staining in wild-type (*n*=6) and *prrx1b*^−/−^ (*n*=7) hearts showing significantly more fibrosis in *prrx1b*^−/−^ hearts inside and around the injury area (mean±s.d., *P*=0.012, unpaired *t*-test).

Next, we mapped the wild-type and *prrx1b*^−/−^ cells separately on the t-distributed stochastic neighbour embedding (tSNE) map to reveal differences in contribution to the various cell clusters (Fig. 4A,B). Interestingly, we found a clear difference in the contribution of wild-type and *prrx1b*^−/−^ cells to the fibroblast clusters 2 and 3. Whereas cluster 2 consisted of mostly wild-type cells (89%) and few *prrx1b*^−/−^ cells (11%), cluster 3 was enriched in *prrx1b*^−/−^ cells (71%) compared with wild-type cells (29%) (Fig. 4B-D). Although both cluster 2 and cluster 3 cells expressed markers for activated fibroblasts, such as *postnb*, differential gene analysis between cluster 2 and 3 revealed that cluster 3 cells are enriched for genes involved in TGFβ signalling (*tgfb1a*, *tgfb2*, *tgfb3*; *P*=7.1E−4), extracellular matrix proteins (*P*=3.3E−4) and collagen fibril organization (*P*=1.2E−4) (Fig. 4E). Instead, cluster 2 cells lacked robust expression of fibrosis-related genes, therefore representing a more quiescent cell type that instead expresses genes linked to chordate embryonic development (*P*=3.8E−10) and stress response (*P*=7.9E−6). Together, the scRNAseq data suggest that injured *prrx1b*^−/−^ hearts contain more activated, pro-fibrotic fibroblasts. To validate these findings, we performed ISH for genes with high expression in cluster 2 or 3 cells. Indeed, we observed increased expression of *tgfb1a* and *col11a1b* in injured *prrx1b*^−/−^ hearts compared with their wild-type siblings (Fig. 4F,G), whereas we identified a strong reduction in expression of the cluster 2 marker *si:dkeyp-1h4.9* (Fig. S7). In addition, we performed Sirius Red staining to visualize collagen, which showed an excess of collagen fibres in the *prrx1b*^−/−^ hearts in and around the injury area (Fig. 4H,I). From these results, we conclude that in injured *prrx1b*^−/−^ hearts an excess of TGFβ ligand and ECM-producing fibroblasts is formed, resulting in an enhanced fibrotic response to the injury.

### NRG1 administration rescues the cardiomyocyte proliferation defect of *prrx1b^−/−^* hearts

**Fig. 5.**
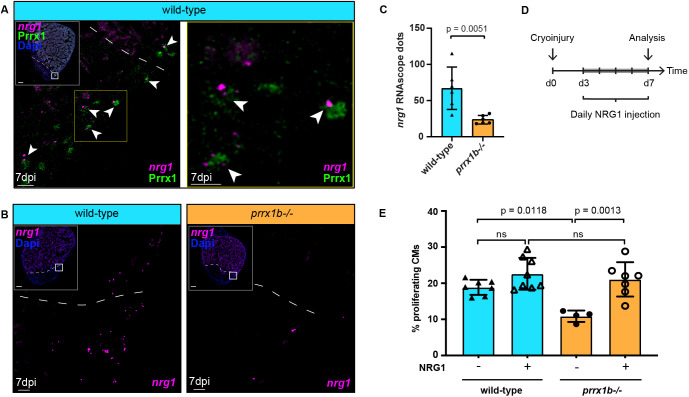
**Prrx1b stimulates Nrg1 expression.** (A) RNAscope *in situ* hybridization for *nrg1* co-detected with Prrx1 antibody on 7 dpi wild-type hearts. Arrowheads indicate colocalization of *nrg1* and Prrx1. Dashed line marks edge of the border zone. Insets show higher magnifications of the boxed areas. Scale bars: 100 μm (main panels); 10 μm (insets). Four hearts analysed. (B) RNAscope *in situ* hybridization for *nrg1* on 7 dpi wild-type and *prrx1b*^−/−^ hearts. Dashed line marks edge of the border zone. Insets show higher magnifications of the boxed areas. Scale bars: 100 μm (main panels); 10 μm (insets). (C) Quantification of *nrg1* RNAscope dots in the BZ epicardium in 7 dpi wild-type (*n*=6) and *prrx1b*^−/−^ (*n*=5) hearts. BZ epicardium is defined as a 100-μm-wide strip, 100 μm up and 100 μm down from where the edge of intact myocardium meets the epicardium (mean±s.d., *P*=0.0051, unpaired *t*-test). (D) Schematic of the workflow used for NRG1 injection experiments shown in E. (E) Quantification of the percentage of proliferating (PCNA^+^) BZ cardiomyocytes (mean±s.d., wild-type −NRG1 *n*=7; wild-type +NRG1 *n*=8; *prrx1b*^−/−^ −NRG1 *n*=4; *prrx1b*^−/−^ +NRG1 *n*=7; wild-type −NRG1 versus *prrx1b*^−/−^ *−*NRG1 *P*=0.0118; *prrx1b*^−/−^ *−*NRG1 versus *prrx1b*^−/−^ *+*NRG1 *P*=0.0013; ns, not significant; one-way ANOVA followed by multiple comparisons analysis using Tukey's test).

Fibroblasts are not only required for fibrosis in the injured zebrafish heart; they also exhibit pro-regenerative activity by stimulating cardiomyocyte proliferation (Sánchez-iranzo et al., 2018). Because EPDCs secrete Nrg1, a growth factor necessary to induce cardiomyocyte proliferation (Gemberling et al., 2015; Ieda et al., 2009; Wang et al., 2015), we hypothesized that *nrg1* expression may be impaired in *prrx1b*^−/−^ hearts and responsible for the observed reduction in cardiomyocyte proliferation. Considering that *nrg1* expression was nearly absent in the scRNAseq data (<100 combined reads from 1438 cells), we investigated expression of *nrg1* through RNAscope ISH. We observed expression of *nrg1* in the epicardial and sub-epicardial region in wild-type hearts at 7 dpi (Fig. 5A,B). The BZ epicardium regions showed profound *nrg1* expression, which is in line with previously reported *nrg1* localization upon injury (Gemberling et al., 2015). Importantly, we observed co-expression of *nrg1* and Prrx1 in BZ epicardial cells at 7 dpi (Fig. 5A). Next, we wanted to investigate whether *nrg1* expression is impaired in *prrx1b*^−/−^ hearts. Corroborating our hypothesis, we found a significant reduction of *nrg1* expression in the BZ epicardium of injured *prrx1b*^−/−^ hearts compared with wild-type sibling hearts (Fig. 5B,C). To address whether the impaired *nrg1* expression in *prrx1b*^−/−^ hearts could explain the observed reduction in cardiomyocyte proliferation, we injected injured wild-type and *prrx1b*^−/−^ fish daily with recombinant NRG1 protein or DMSO as a control from 3 dpi to 7 dpi and quantified cardiomyocyte proliferation in the border zone. Importantly, injecting *prrx1b*^−/−^ zebrafish with recombinant NRG1 protein did indeed rescue cardiomyocyte proliferation in the border zone to wild-type levels (Fig. 5D,E). Together, these results demonstrate that *nrg1* and Prrx1 are co-expressed and that Prrx1 promotes *nrg1* expression in EPDCs. Furthermore, the results suggest that the reduction in Nrg1 is responsible for the reduced cardiomyocyte proliferation observed in the border zones of *prrx1b* mutant hearts.

### PRXX1 promotes NRG1 expression in human EPDCs

**Fig. 6.**
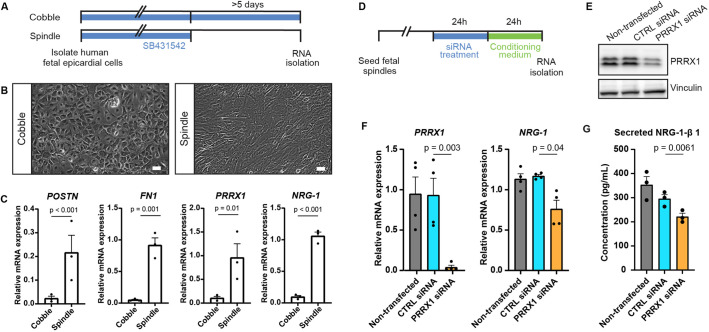
**PRRX1 promotes NRG1 expression in human EPDCs.** (A) Schematic of the workflow for the experiments shown in B and C. After isolation, human fetal epicardial cells are cultured in the presence of the ALK4/5/7 kinase inhibitor SB-431542. Cells transform from cobble- to spindle-shape upon removal of SB-431542. (B) Representative brightfield pictures of cobble- and spindle-shaped human fetal epicardial cells. Scale bars: 100 µm. (C) qPCR results for *POSTN*, *FN1*, *PRRX1* and *NRG1* in human fetal cobble (*n*=3) and spindle (*n*=3) epicardial cells (mean±s.d.; *POSTN P*<0.001, *FN1 P*=0.001, *PRRX1 P*=0.01, *NRG1 P*<0.001, unpaired *t*-tests). (D) Schematic of the workflow for the experiments shown in E and F. (E) Western blot for PRRX1 in U87 cells. Vinculin was used as a loading control. (F) qPCR results for *PRRX1* and *NRG1* in human fetal spindle epicardial cells after PRRX1 siRNA treatment (non-transfected cells *n*=4, CTRL siRNA *n*=4, PRRX1 siRNA *n*=4) (mean±s.d., PRRX1 CTRL siRNA versus PRRX1 siRNA *P*=0.003, NRG1 CTRL siRNA versus PRRX1 siRNA *P*=0.04, unpaired *t*-tests) (G) ELISA results for secreted NRG1-β1 in the conditioned cell culture medium of human fetal spindle epicardial cells between 24 and 48 h after PRRX1 siRNA treatment (non-transfected cells *n*=3, CTRL siRNA *n*=3, PRRX1 siRNA *n*=3) (mean±s.d., CTRL siRNA versus PRRX1 siRNA *P*=0.0061, unpaired *t*-tests).

Next, we wanted to address whether *nrg1* expression in EPDCs is regulated by Prrx1. As the *prrx1b*^−/−^ fish lack Prrx1 in all cells, we exploited a previously established *in vitro* model (Dronkers et al., 2018) in which human fetal epicardial cells can be cultured in an epithelial phenotype in the presence of the ALK4/5/7 kinase inhibitor SB-431542. Removal of the inhibitor for at least 5 days results in the induction of EMT, which can be appreciated by the transition of cobblestone epithelial-like cells towards spindle-shaped mesenchymal cells and upregulation of the mesenchymal genes *POSTN* and *FN1* (Fig. 6A-C) (Moerkamp et al., 2016). Although some *PRRX1* expression was detected in cobblestone epithelial-like cells, its expression was increased 8-fold in spindle-shaped mesenchymal cells (Fig. 6C). Importantly, *NRG1* expression followed the same pattern as *PRRX1* expression, as they were both increased in spindle-shaped cells. To determine whether PRRX1 can regulate *NRG1* expression, spindle-shaped mesenchymal cells were subjected to *PRRX1* knockdown (KD) using siRNAs (Fig. 6D). The effect of PRRX1 KD was confirmed using western blot (Fig. 6E). Indeed, *PRRX1* KD led to a significant decrease in *NRG1* mRNA, as well as a significant decrease of secreted NRG1-β1 protein (Fig. 6F,G). From these results, we conclude that in EPDCs after EMT induction, PRRX1 and NRG1 are co-expressed and that PRRX1 is required for efficient NRG1 expression.

## DISCUSSION

The results described here demonstrate that *prrx1b* is required for the scar-free regeneration of the injured zebrafish heart. The zebrafish genome contains a *prrx1a* and a *prrx1b* gene, which are likely the result of an ancient genome duplication that occurred in teleosts (Howe et al., 2013). Our results demonstrate that whereas *prrx1b* is required for heart regeneration, *prrx1a* is dispensable, which suggests these paralogues have non-redundant roles. This is different from their role during cartilage formation in the embryo where *prrx1a* and *prrx1b* are redundant (Barske et al., 2016).

Prrx1 expression is rapidly induced in the epicardium upon injury. This is reminiscent of the induction of other genes in the epicardium, such as *tbx18* and *aldh1a2* (also known as *raldh2*), and implies that Prrx1 induction is part of the early activation that occurs in the entire epicardium (Cao and Poss, 2018; Lepilina et al., 2006). Importantly, all three previously identified subpopulations (*tcf21*^+^, *tbx18^+^ and wt1^+^*) of epicardial cells and EPDCs express Prrx1 (Cao et al., 2016; Weinberger et al., 2020).

It has been well established that EPDCs differentiate into various cell types (reviewed by Cao and Poss, 2018). Retroviral labelling and Cre-mediated recombination studies in chick, mouse and zebrafish have demonstrated that EPDCs differentiate into fibroblasts and vascular support cells (e.g. pericytes) (Acharya et al., 2012; Gittenberger-de Groot et al., 1998; González-Rosa et al., 2012; Kikuchi et al., 2011; Männer, 1999; Mikawa and Gourdie, 1996), which is in good agreement with our scRNAseq data. There are also numerous reported observations suggesting that EPDCs can differentiate into endothelial cells and cardiomyocytes (Cai et al., 2008; Guadix et al., 2006; Katz et al., 2012; Männer, 1999; Mikawa and Gourdie, 1996; Smart et al., 2011; Zangi et al., 2013), although some of these observations have been questioned by others (Christoffels et al., 2009; Rudat and Kispert, 2012). In our scRNAseq analysis of EPDCs recovered from the regenerating zebrafish heart, we did not find a cell type representing endothelial cells or cardiomyocytes, which is in agreement with earlier observations that *tcf21*-derived EPDCs in the zebrafish do not contribute to either endothelial or myocardial cell lineages (González-Rosa et al., 2012; Kikuchi et al., 2011).

Fibroblasts are one of the main contributors to ECM deposition in response to cardiac injury and are therefore an important cell type in maintaining the balance between the fibrotic and regenerative injury response (Chablais and Jaźwińska, 2012; Gemberling et al., 2015; Sánchez-iranzo et al., 2018). In addition, subpopulations of cardiac fibroblasts can have distinct roles in in cardiomyocyte maturation and innervation (Hortells et al., 2020). By scRNAseq analysis, we identified two distinct fibroblast cell states in the regenerating heart. The pro-fibrotic fibroblast cluster (cluster 3) expresses all three TGFβ ligands, supporting earlier findings that these ligands are expressed in the injury area to activate a pro-fibrotic response (Chablais and Jaźwińska, 2012). Pro-fibrotic fibroblasts express fibronectin-1 (*fn1a*) and various collagens (Sánchez-iranzo et al., 2018), which we found to be upregulated in the cluster 3 fibroblasts, corroborating their pro-fibrotic nature. In *prrx1b*^−/−^ hearts, these pro-fibrotic fibroblasts were more abundant, which is consistent with the observed excess of collagen deposition. Whereas cardiac fibrosis is permanent in the injured mammalian heart, it is resolved in the zebrafish heart. The mechanism for this regression in the zebrafish heart is not well understood. It could be related to the observation that activated fibroblasts partially return to a quiescent state (Sánchez-iranzo et al., 2018). Our results showing that an increase in activated (pro-fibrotic) fibroblast cell numbers can lead to an excessive fibrotic response supports the theory that the de-activation of injury-responsive pro-fibrotic fibroblasts is detrimental to successful scar regression.

Cluster 2 cells have only limited expression of pro-fibrotic genes and might therefore represent de-activated, quiescent fibroblasts. Many factors secreted by activated fibroblasts have been implicated in cardiac development and regeneration, suggesting that the pro-regenerative function of fibroblasts might be accomplished through their secretory role. In addition, experiments co-culturing fibroblasts with cardiomyocytes show that fibroblasts can induce cardiomyocyte proliferation (Ieda et al., 2009). Our results demonstrate Prrx1-dependent *nrg1* expression in EPDCs near the proliferating cardiomyocytes in the border zone. Nrg1 is a potent inducer of cardiomyocyte proliferation by activation of the ErbB2 signalling pathway (Bersell et al., 2009; D'Uva et al., 2015; Gemberling et al., 2015), which is consistent with our observation that the cardiomyocyte proliferation defect in *prrx1b*^−/−^ hearts can be rescued by exogenous Nrg1. Both the *in vitro* experiments in human fetal EPDCs and the *in vivo* experiments in zebrafish demonstrate that *Nrg1* expression depends on Prrx1. Whether this is through binding of Prrx1 to regulatory sequences in the *nrg1* locus or whether is through a more indirect mechanism needs to be further investigated for example by chromatin immunoprecipitation experiments.

In conclusion, we have shown that during zebrafish heart regeneration Prrx1b expression in EPDCs restricts the amount of pro-fibrotic fibroblasts and stimulates cardiomyocyte proliferation. In doing so, Prrx1b establishes a balance between fibrotic repair and the regeneration of lost myocardium during zebrafish heart regeneration.

## MATERIALS AND METHODS

### Animal experiments

Animal care and experiments conformed to the Directive 2010/63/EU of the European Parliament. All animal work was approved by the Animal Experimental Committee of the Instantie voor Dierenwelzijn Utrecht (IvD) and was performed in compliance with the Dutch government guidelines. Zebrafish were housed under standard conditions (Aleström et al., 2019).

### Zebrafish lines

The following zebrafish lines were used: TL, *prrx1a*, *prrx1a^el558^*, *prrx1b^el491^* (Barske et al., 2016), *Tg(tcf21:CreERT2)* (Kikuchi et al., 2011) and *Tg(ubi:loxP-EGFP-loxP-mCherry)* (Mosimann et al., 2011).

### Cryoinjuries in zebrafish

To address experiments in a regeneration context, cardiac cryo-injuries were performed on TL and *prrx1b^el491^* [with and without *Tg(tcf21:CreERT2; ubi:loxP-EGFP-loxP-mCherry)*] fish of both sexes that were ∼4-18 months of age. The cryoinjuries were performed as described by Schnabel et al. (2011), with the exception of the use of a copper filament (0.3 mm) cooled in liquid nitrogen instead of dry ice. Animals were excluded from the study if they exhibited signs of aberrant behaviour, sickness or infection, according to animal care guidelines.

### Histology and enzyme histochemistry

Acid Fuchsin Orange G (AFOG) staining was performed on paraffin sections of zebrafish ventricles as previously described (Poss et al., 2002). Paraffin sections of 7, 30 and 90 dpi hearts were prepared as described below (see ‘*In situ* hybridization’ section). Sirius Red staining was performed on similar paraffin sections as previously described (Junqueira et al., 1979), excluding the Haematoxylin step.

### Immunofluorescence

Adult zebrafish ventricles were isolated and fixed in 4% paraformaldehyde (4°C overnight on shaker). The next day, the hearts were washed three times, 10 min each wash, in 4% sucrose phosphate buffer, after which they were incubated at room temperature for at least 5 h in 30% sucrose phosphate buffer until the hearts sank. Then, they were embedded in cryo-medium (OCT). The hearts were cryosectioned at 10 μm thickness using a Thermo Scientific Cryostar NX70 cryostat. Primary antibodies used were: anti-PCNA (Dako, M0879; 1:800), anti-Mef2c (Santa Cruz Biotechnology, SC313 or Biorbyt, orb256682; both 1:1000), anti-tropomyosin (Sigma-Aldrich, 122M4822; 1:400), Living Colors anti-DsRed (Clontech, 632496; 1:100), anti-RFP (Novus Biologicals, 42649; 1:100), anti-Prrx1 (gift from the Tenaka lab; Gerber et al., 2018; Oliveira et al., 2017; 1:200), mouse IgG2b anti-Dendra2 [Origene, TA180094, clone OTI1G6 (for Wt1b H2B dendra); 1:400], chicken polyclonal anti-GFP [Abcam, ab13970 (for Tbx18 myr GFP); 1:200]. Secondary antibodies were: anti-chicken Alexa 488 (Thermo Fisher, A21133; 1:500), anti-rabbit Alexa 555 (Thermo Fisher, A21127; 1:500), anti-mouse Cy5 (Jackson ImmunoResearch, 118090; 1:500), anti-mouse IgG2b Alexa 647 (Jackson ImmunoResearch, 102371; 1:100). Nuclei were stained using 4′,6-diamidino-2-phenylindole (DAPI) or Hoechst 405 staining. Images of immunofluorescence staining are single optical planes acquired with a Leica SP8 confocal microscope.

### Quantitative analyses

All quantifications were performed blind. Unless stated otherwise, three individual sections with the largest injuries per heart were analysed including data obtained through *in situ* hybridization, immunohistochemistry and Sirius Red staining. Imaris x64 V3.2.1 software (Oxford Instruments) was used to analyse immunofluorescence images made with a Leica SP8 confocal microscope. Proliferation percentages of border zone cardiomyocytes were determined using the spots selection tool in Imaris. A region of interest (200 μm) consisting of the border zone was chosen and cardiomyocytes (identified by Mef2 expression) were selected by classifying them as 5 μm diameter or bigger. Proliferating cardiomyocytes were selected by hand using the PCNA channel. To quantify the distribution of *tcf21*^+^ Prrx1^+^ cells over different locations and different time points, the spot selection tool in Imaris was used to select all Prrx1^+^ cells in the ventricle, after which a subselection of all *tcf21:*mCherry^+^ Prrx1^+^ cells was made by hand. Distinguished regions were remote epicardium, BZ epicardium (100 μm up and 100 μm down from the edge of intact myocardium), injury epicardium and within the injury. Double-positive cells in each of these regions were counted and presented as a percentage of the total double-positive cells in the ventricle. To quantify the percentage of *tcf21:*mCherry^+^ cell invasion into the injury, the surface selection tool was used to mark the total *tcf21:*mCherry^+^ area in the ventricle. The measurement we used was the average value of the volume. Then, the total injury area plus 100 μm border zone was chosen as a region of interest designated as the ‘whole injury area’. Then, *tcf21:*mCherry^+^ surfaces within the injury were selected manually to create a subset of the whole injury area surface. Proliferation of *tcf21:*mCherry^+^ cells was defined as the number of PCNA^+^ cells per μm^2^ of *tcf21:*mCherry^+^ tissue surface, as the cytoplasmic mCherry signal does not allow for the distinction between individual cells. *tcf21:*mCherry^+^ PCNA^+^ cells were counted manually. *Nrg1* RNAscope signal was quantified by using the spots selection tool in Imaris to count the absolute number of *nrg1* transcripts in the BZ epicardium regions. ImageJ software (NIH) was used to quantify the remaining scar size of 30 and 90 dpi heart sections following AFOG staining. All sections of each heart were stained, imaged and quantified for scar tissue area using ImageJ. Sirius Red staining in wild-type and *prrx1b*^−/−^ hearts was analysed using the ImageJ-macros MRI Fibrosis Tool (http://dev.mri.cnrs.fr/projects/imagej-macros/wiki/Fibrosis_Tool).

### Lineage tracing of zebrafish epicardial cells

To lineage trace epicardial and epicardial-derived cells, we combined *Tg(tcf21:CreERT2)* with *Tg(ubi:loxP-EGFP-loxP-mCherry)*. Both wild-type and *prrx1b*^−/−^ embryos with a single copy of both transgenes [*Tg(tcf21:CreERT2; ubi:loxP-EGFP-loxP-mCherry)*] were incubated in 4-hydroxytamoxifen (4-OHT) as described by Kikuchi et al. (2011) and Mosimann et al. (2011) from 1 dpf until 5 dpf at a concentration of 5 µM. At 5 dpf, embryos were selected that were positive for epicardial mCherry signal and grown to adulthood.

### Isolation of single cells from cryoinjured hearts

Cryoinjured hearts of either *prrx1b* wild-type siblings (*n*=20) or *prrx1b* homozygous mutants (*n*=20) previously recombined as embryos [*Tg(tcf21:CreERT2; ubi:loxP-EGFP-loxP-mCherry*] were extracted at 7 dpi. Cells were dissociated according to Tessadori et al. (2012). For cell sorting, viable cells were gated by negative DAPI staining and positive YFP fluorescence. In brief, the FACS gating was adjusted to sort cells positive for mCherry (recombined epicardial-derived cells) and negative for EGFP (unrecombined cells). In total, 1536 cells (768 *prrx1b* wild-type sibling cells and 768 *prrx1b* homozygous mutant cells) were sorted into 384-well plates and processed for scRNAseq as described below.

### scRNAseq

Single-cell sequencing libraries were prepared using SORT-seq (Muraro et al., 2016). Live cells were sorted into 384-well plates with Vapor-Lock oil containing a droplet with barcoded primers, spike-in RNA and dNTPs, followed by heat-induced cell lysis and cDNA syntheses using a robotic liquid handler. Primers consisted of a 24 bp polyT stretch, a 4 bp random molecular barcode (UMI), a cell-specific 8 bp barcode, the 5′ Illumina TruSeq small RNA kit adapter and a T7 promoter. After cell lysis for 5 min at 65°C, RT and second strand mixes were distributed with the Nanodrop II liquid handling platform (Inovadyne). After pooling all cells in one library, the aqueous phase was separated from the oil phase, followed by IVT transcription. The CEL-Seq2 protocol was used for library prep (Hashimshony et al., 2016). Illumina sequencing libraries were prepared with the TruSeq small RNA primers (Illumina) and paired-end sequenced at 75 bp read length on the Illumina NextSeq platform. Mapping was performed against the zebrafish reference assembly version 9 (Zv9).

### Bioinformatic analysis

To analyse the scRNAseq data, we used an updated version (RaceID3) of the previously published RaceID algorithm (Grün et al., 2015). For the adult hearts, we obtained a dataset consisting of two different libraries of 384 cells per genotype (wild type or *prrx1b* homozygous mutants) each for a combined dataset of 768 cells, in which we detected a total of 20,995 genes. We detected an average of 7022 reads per cell. Based on the distribution of the log10 total reads plotted against the frequency, we introduced a cutoff at minimally 1000 reads per cell before further analysis. This reduced the number of cells used in the analysis to 711 wild-type and 727 mutant cells. The top 20 noisy genes were identified by the StemID algorithm, which we excluded from the downstream analysis to increase clustering robustness. Batch-effects were analysed and showed no plate-specific clustering of certain clusters. The StemID algorithm were used as previously described (Grün et al., 2016). In short, StemID is an approach developed for inferring the existence of stem cell populations from single-cell transcriptomics data. StemID calculates all pairwise cell-to-cell distances (1 – Pearson correlation) and uses this to group similar cells into clusters that correspond to the cell types present in the tissue. The StemID algorithm calculates the number of links between clusters. This is based on the assumption that cell types with fewer links are more canalized whereas cell types with a higher number of links have a higher diversity of cell states. Besides the number of links, the StemID algorithm also calculates the change in transcriptome entropy. Differentiated cells usually express a small number of genes at high levels in order to perform cell-specific functions, which is reflected by a low entropy. Stem cells and progenitor cells display a more diverse transcriptome reflected by high entropy (Banerji et al., 2013). By calculating the number of links from one cluster to other clusters and multiplying this with the change in entropy, it generates a StemID score, which is representative of the ‘stemness’ of a cell population. Differential gene expression analysis was performed using the ‘diffexpnb’, which makes use of the DESeq algorithm. *P*-values were Benjamini–Hochberg corrected for false discovery rate to make the cutoff.

### Statistical analysis of data

Statistical analyses were performed using GraphPad Prism8 software. Unless stated otherwise, unpaired *t*-tests were used for all statistical testing. For the NRG1 rescue experiment (Fig. 5E) one-way ANOVA followed by multiple comparisons analysis using the Tukey's test was performed.

### *In situ* hybridization

*In situ* hybridization was performed on paraffin sections. After overnight fixation in 4% paraformaldehyde, hearts were washed in PBS twice, dehydrated in ethanol, and embedded in paraffin. Serial sections were made at 8 μm thickness. *In situ* hybridization was performed as previously described (Moorman et al., 2001) except that the hybridization buffer used did not contain heparin and yeast total RNA. When *in situ* hybridization was carried out for multiple probes, INT-BCIP staining solution (red/brown staining) was used for the additional probe instead of NBT-BCIP (blue staining).

### RNAscope

RNAscope *in situ* hybridization was performed on fixed frozen sections following the Advanced Cell Diagnostics company protocol for RNAscope Multiplex Fluorescent Reagent Kit v2 with the following modification: target retrieval was not performed as this was not required for the *nrg1* probe. The probe used for *nrg1* detection was RNAscope Probe- Dr-nrg1-CDS (414131). For co-detection with Prrx1 antibody, the RNA-Protein Co-detection Ancillary Kit was used following the Advanced Cell Diagnostics company protocol. Prrx1 antibody was used at 1:200.

### Intraperitoneal injections in zebrafish

Intraperitoneal injections of human recombinant NRG1 (recombinant human heregulin-b1, Peprotech, 100-03) were performed as described by Kinkel et al. (2010). Fish were sedated using MS222 (0.032% wt/vol). Injections were performed using a Hamilton syringe (Gauge 30), cleaned before use by washing in 70% ethanol followed by two washes in PBS. Injection volumes were adjusted to the weight of the fish (30 μl/g) and a single injection contained 60 μg/kg (diluted in 0.1% bovine serum albumin in PBS).

### Human epicardial cell culture

Human fetal hearts of a gestational age between 12 and 18 weeks were collected anonymously and under informed consent from abortion material after elective abortion. Epicardial cells were isolated as described by Dronkers et al. (2018). Cells were cultured in Dulbecco's modified Eagle's medium (DMEM low-glucose, Gibco) and Medium 199 (M199, Gibco) mixed in a 1:1 ratio, supplemented with 10% fetal bovine serum (heat-inactivated for 25 min at 56°C, Gibco), 100 U/ml penicillin (Roth), 100 mg/ml streptomycin (Roth) and 10 µM ALK4/5/7 kinase inhibitor SB-431542 (Tocris) at 37°C in 5% CO_2_. EMT was induced by removal of SB-431542 from the medium. This research was carried out according to the official guidelines of the Leiden University Medical Center and approved by the local Medical Ethics Committee. This research conforms to the Declaration of Helsinki. Cells were tested for contamination every 3 months.

### Cell culture U87 cells

U87 cells were cultured in Dulbecco's modified Eagle's medium (DMEM high-glucose, Gibco), supplemented with 10% fetal bovine serum (Gibco), 100 U/ml penicillin (Roth) and 100 mg/ml streptomycin (Roth). Cells were tested for contamination every 3 months.

### PRRX1 KD in human epicardial cells

Cells were treated with SMARTpool ON-TARGETplus PRRX1 or a non-targeting control siRNA according to the manufacturer's protocol at a concentration of 25 nM (Dharmacon). After 48 h, cells were collected for qPCR or western blot. All experiments in human fetal epicardial cells were performed with three or four individual cell isolations.

### qPCR

ReliaPrep RNA Miniprep Systems (Promega) was used to isolate mRNA, of which the concentration and purity were determined using a NanoDrop 1000 Spectrophotometer (Thermo Fisher Scientific). cDNA synthesis was performed using the RevertAid H Minus First Strand cDNA Synthesis Kit (Thermo Fisher Scientific). Next, qPCR was performed using SYBR Green (Promega) and run on a C1000 Touch thermal cycler (Bio-Rad). All samples were run in triplicate; expression levels were corrected for primer efficiency and normalized for two reference genes (*TBP* and *HPRT1*).

Primer sequences were: *POSTN* forward, GGAGGCAAACAGCTCAGAGT; *POSTN* reverse, GGCTGAGGAAGGTGCTAAAG; *FN1* forward, CGTCATAGTGGAGGCACTGA; *FN1* reverse, CAGACATTCGTTCCCACTCA; *PRRX1a* forward, CGCAGGAATGAGAGAGCCAT; *PRRX1a* reverse, AACATCTTGGGAGGGACGAG; *NRG1* forward, CACATGATGCCGACCACAAG; *NRG1* reverse, GGTGATCGCTGCCAAAACTA; *TBP* forward, TGGAAAAGTTGTATTAACAGGTGCT; *TBP* reverse, GCAAGGGTACATGAGAGCCA; *HPRT1* forward, CTCATGGACTGATTATGGACAGGAC; *HPRT1* reverse, GCAGGTCAGCAAAGAACTTATAGCC.

### Western blot

Cells were lysed in radioimmunoprecipitation assay (RIPA) buffer containing protease and phosphatase inhibitors (cOmplete Protease Inhibitor Cocktail tablets, Roche Diagnostics). Protein concentration was determined using the Pierce BCA Protein Assay Kit (Thermo Fisher Scientific). For every sample, 50 μg of protein was loaded onto a 10% SDS-polyacrylamide gel. Subsequently, protein was transferred onto Immobilon-P PVDF Membrane (Millipore). Blots were blocked in 5% bovine serum albumin in Tris-buffered saline with 0.1% Tween 20 (TBST) for 1 h and incubated overnight with primary antibody (anti-PRRX1, 1:500, gift from the Tenaka lab; Gerber et al., 2018; Oliveira et al., 2017; anti-Vinculin, 1:5000, Sigma-Aldrich, V9131, 1:5000). Blots were incubated for 1 h with horseradish peroxidase anti-rabbit secondary antibody (Abcam, ab98493), which was detected by WesternBright Quantum (Advansta).

### ELISA

Conditioned medium was collected for 24 h after 24 h of siRNA treatment, centrifuged at 200 ***g*** for 2 min and immediately frozen at −20°. Cell culture medium was taken as a control sample. An NRG1-β1 ELISA assay was performed according to the manufacturer's protocol (Human NRG1-β1 DuoSet ELISA, R&D Systems). Absolute NRG1-β1 concentration was calculated based on the standard curve.

## Supplementary Material

Supplementary information

Reviewer comments
