# Supplementary material for: Prrx1b restricts fibrosis and promotes Nrg1-dependent cardiomyocyte proliferation during zebrafish heart regeneration
Source: Development. 2021 Oct 4;148(19):dev198937. doi: 10.1242/dev.198937 (PMC8513610; doi:10.1242/dev.198937)
Supplement: Supplementary information [file develop-148-198937-s1.pdf]

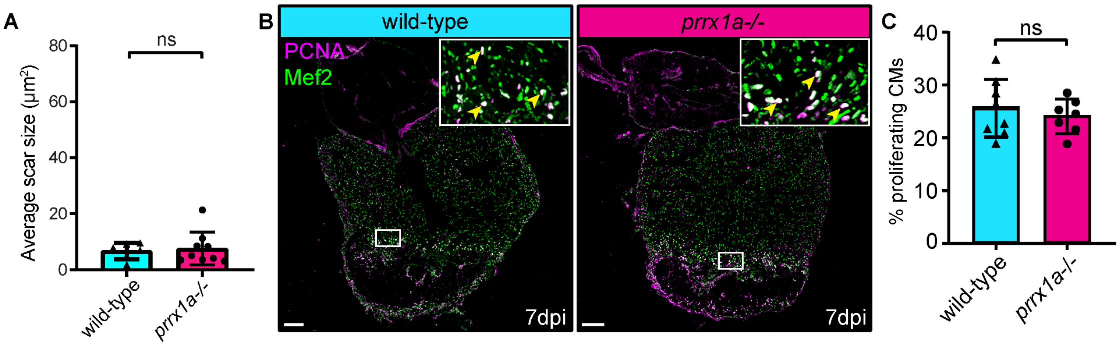

Fig. S1. *prrx1a* is dispensable for zebrafish border zone cardiomyocyte proliferation and heart regeneration. (A) Quantification of the remaining scar size at 30dpi shows no significant difference between *prrx1a-/-* hearts (n=9) and wild-type siblings (n=6). (mean±s.d., ns= not significant, unpaired t-test). **(B)** Immunofluorescent staining on 7dpi wild-type and *prrx1a-/-* heart sections using an anti-Mef2 antibody as a marker for cardiomyocyte nuclei, and an anti-PCNA antibody as a nuclear proliferation marker. Arrowheads in zoom-ins indicate proliferating cardiomyocytes. Scale bars represent 100µm in the overview images and 10µm in the zoom-ins. **(C)** Quantification of the percentage of (PCNA+) proliferating border zone cardiomyocytes shows no significant difference between *prrx1a-/-* hearts (n=7) and their wild-type siblings (n=8). (mean±s.d., ns= not significant, unpaired t-test).

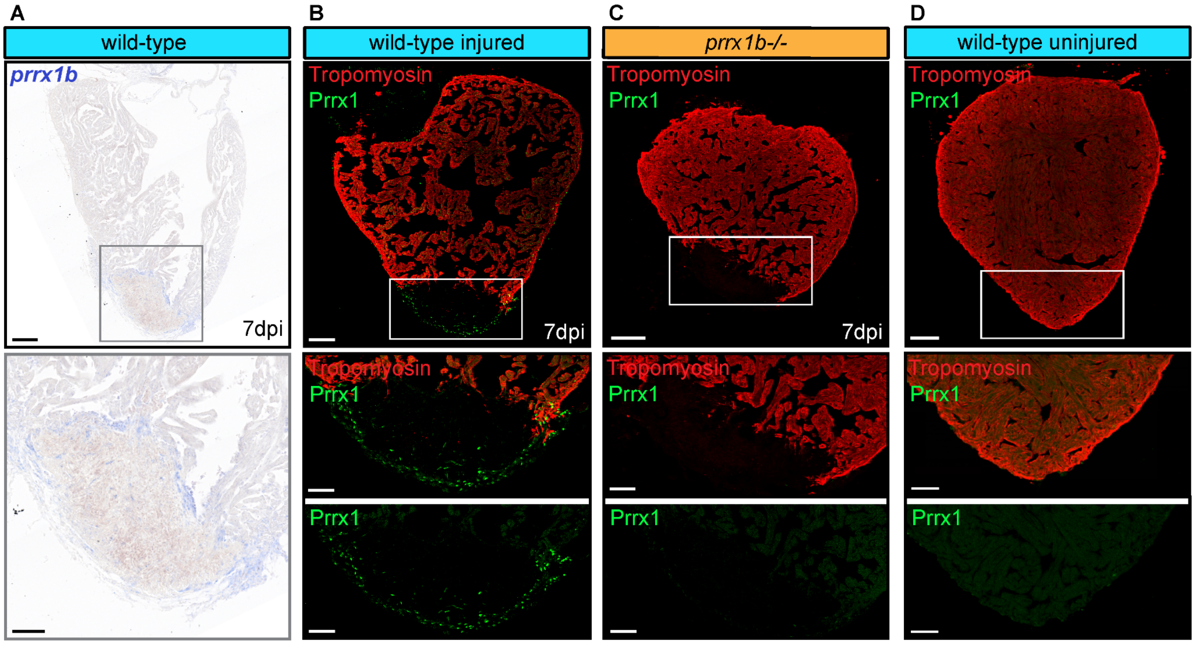

Fig. S2. Injured zebrafish hearts express Prrx1 in cells surrounding the injury area, which is severely reduced in the *prrx1b*-/- hearts. (A) *in situ* hybridization for *prrx1b* on 7dpi wild-type hearts shows *prrx1b* mRNA surrounding and within the injury area. (**B-D**) Immunofluorescent staining of tropomyosin staining CM nuclei (red) and Prrx1 protein (green) in (**B**) injured wild-type hearts at 7dpi, (**C**) injured *prrx1b*-/- hearts at 7 dpi and (**D**) uninjured wildtype hearts. Scale bars represent 100µm in the overview images and 50µm in the zoom-ins. Hearts analyzed per condition: 3.

Development • Supplementary information

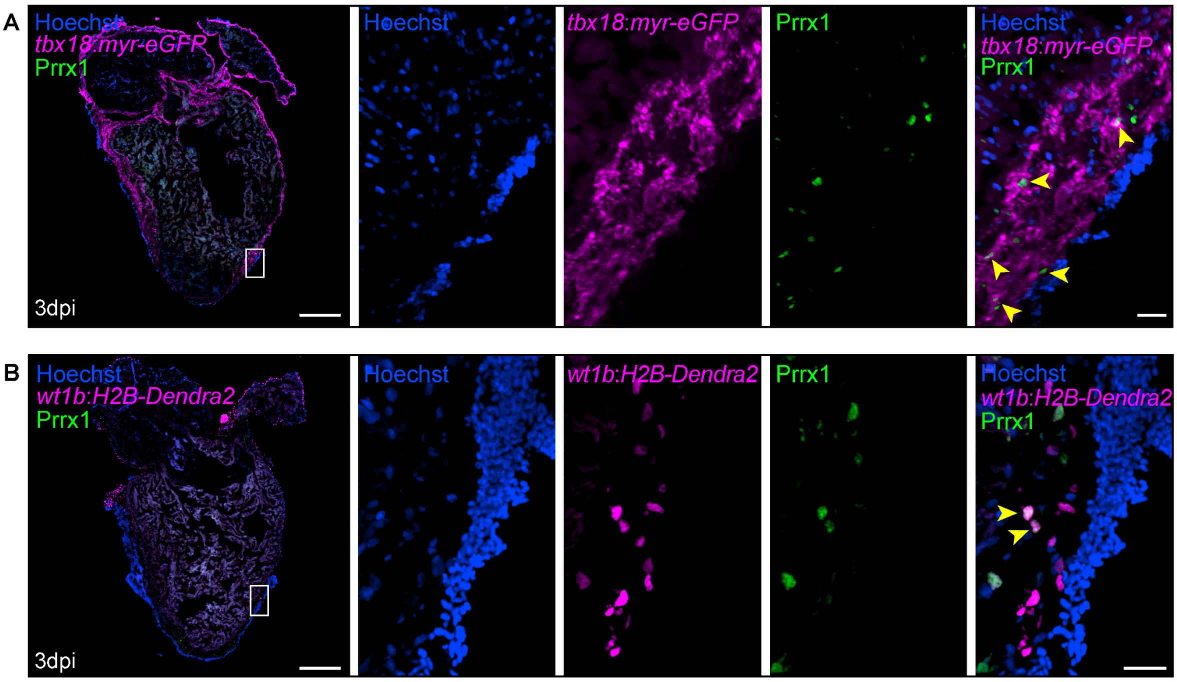

Fig. S3. Prrx1 is expressed in *tbx18*⁺ and *wt1b*⁺ epicardial cells. **(A)** Immunofluorescent staining on a 3dpi Tg(*tbx18:myr-eGFP*) heart, staining Hoechst, *tbx18:myr eGFP* (membrane) and Prrx1 (nuclei). Arrowheads indicate double positive cells. **(B)** Immunofluorescent staining on a 3dpi Tg(*wt1b:H2B-Dendra2*) heart, staining Hoechst, *wt1b:H2B-Dendra2* (nuclei) and Prrx1 (nuclei). Arrowheads indicate double positive cells. Scale bars in the overview images represent 100um and in the zoom ins 20um.

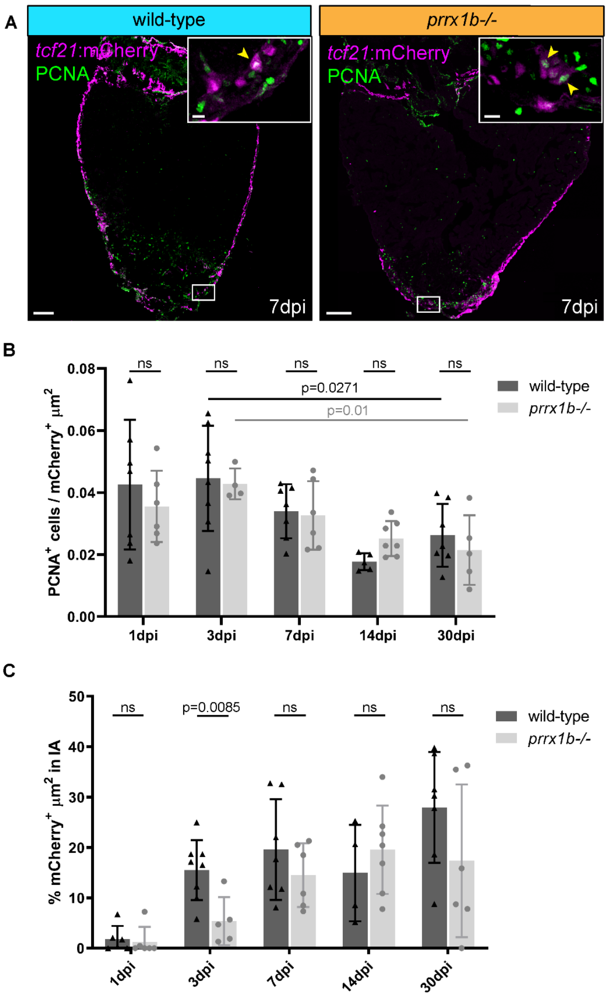

Fig. S4. Quantification of invasion and proliferation of *tcf21:mCherry*+ cells at 1, 3, 7, 14 and 30dpi in wild-type and *prrx1b-/-* hearts. (A) Immunofluorescent staining showing PCNA (green) and *tcf21:mCherry* (magenta) in wild-type sibling and *prrx1b-/-* hearts at 7dpi. Scalebar = 100µm, scale bar in zoom in = 10µm. **(B)** Proliferation of *tcf21:mCherry*+ cells quantified as amount of PCNA+ cells per total µm^2 *of tcf21:mCherry*+ tissue surface in the ventricle at 1, 3, 7, 14 and 30dpi. (mean±s.d., wild-type 3dpi vs wild-type 30dpi p=0.0271, *prrx1b-/-* 3dpi vs *prrx1b-/-* 30dpi p=0.01, ns= not significant, unpaired t-test). **(C)** Percentage of the total injury *tcf21:mCherry*+ µm2 found inside the injury area at 1, 3, 7, 14 and 30dpi. (mean±s.d., wild-type 3dpi vs *prrx1b-/-* 3dpi p=0.0085, ns=not significant, unpaired t-test). IA = injury area.

Development • Supplementary information

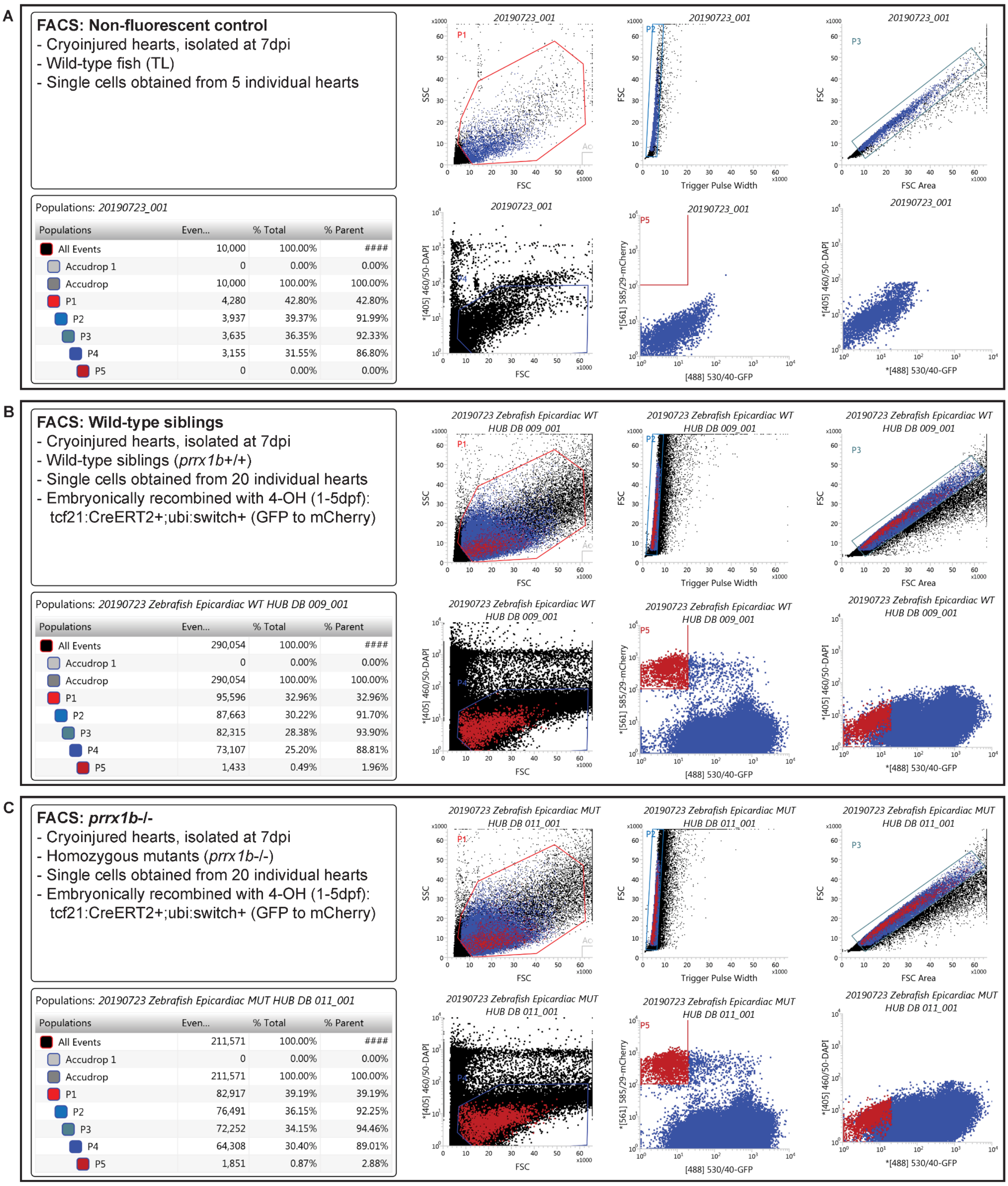

Populations: 20190723_001

Populations	Even...	% Total	% Parent
All Events	10,000	100.00%	####
Accudrop 1	0	0.00%	0.00%
Accudrop	10,000	100.00%	100.00%
P1	4,280	42.80%	42.80%
P2	3,937	39.37%	91.99%
P3	3,635	36.35%	92.33%
P4	3,155	31.55%	86.80%
P5	0	0.00%	0.00%

Populations: 20190723 Zebrafish Epicardiac WT HUB DB 009_001

Populations	Even...	% Total	% Parent
All Events	290,054	100.00%	####
Accudrop 1	0	0.00%	0.00%
Accudrop	290,054	100.00%	100.00%
P1	95,596	32.96%	32.96%
P2	87,663	30.22%	91.70%
P3	82,315	28.38%	93.90%
P4	73,107	25.20%	88.81%
P5	1,433	0.49%	1.96%

Populations: 20190723 Zebrafish Epicardiac MUT HUB DB 011_001

Populations	Even...	% Total	% Parent
All Events	211,571	100.00%	####
Accudrop 1	0	0.00%	0.00%
Accudrop	211,571	100.00%	100.00%
P1	82,917	39.19%	39.19%
P2	76,491	36.15%	92.25%
P3	72,252	34.15%	94.46%
P4	64,308	30.40%	89.01%
P5	1,851	0.87%	2.88%

Fig. S5. Gating details for FACS. (A-C) Gates (P1-P5) used to sort out mCherry$^+$/GFP$^-$ cells used for single cell sequencing analysis.

Development • Supplementary information

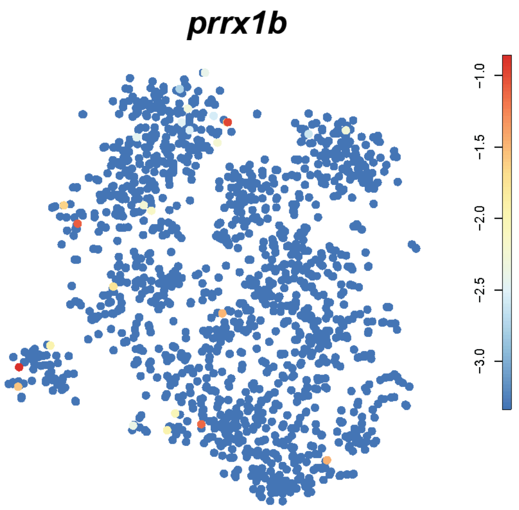

Fig. S6. *prrx1b* read counts. tSNE map visualizing log2-transformed read-counts for *prrx1b* based on our scRNA sequencing of *tcf21:mCherry+* cells.

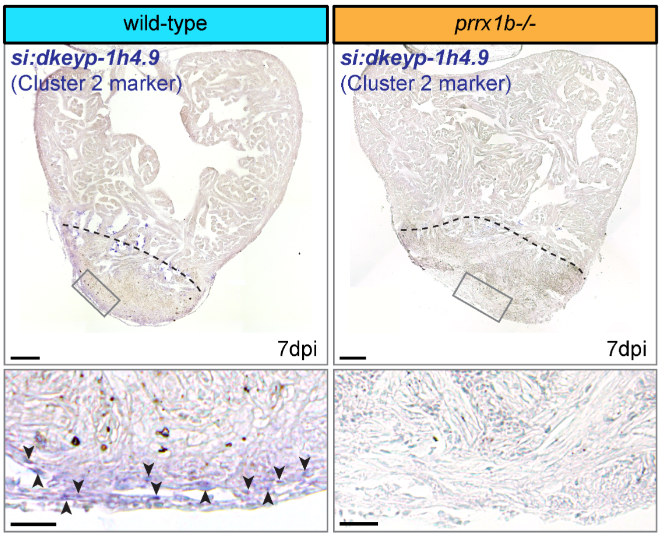

Fig. S7. *prrx1b-/-* hearts show a strong reduction of si:dkeyp-1h4.9 expression. (A,B) *In situ* hybridization for the cluster 2 gene si:dkeyp-1h4.9 in either wild-type or *prrx1b-/-* hearts at 7dpi. Arrowheads point to cells with high expression. Scale bars represent 100µm in the overview images and 25µm in the zoom-in. Hearts analysed per condition: 3

Development • Supplementary information

Table S1. Differentially expressed genes for scRNAseq cluster identification

Click here to download Table S1

Table S2. Differentially expressed genes in multiple scRNAseq clusters

Click here to download Table S2

Table S3. Gene Ontologies for differentially expressed genes in multiple scRNAseq clusters

Click here to download Table S3
