# Supplementary material for: Prrx1b restricts fibrosis and promotes Nrg1-dependent cardiomyocyte proliferation during zebrafish heart regeneration
Source: Development. 2021 Oct 4;148(19):dev198937. doi: 10.1242/dev.198937 (PMC8513610; doi:10.1242/dev.198937)
Supplement: Reviewer comments [file develop-148-198937_review_history.pdf]

Prrx1b restricts fibrosis and promotes Nrg1-dependent cardiomyocyte proliferation during zebrafish heart regeneration

Dennis E.M. de Bakker, Mara Bouwman, Esther Dronkers, Filipa C. Simões, Paul R. Riley, Marie-José Goumans, Anke M. Smits and Jeroen Bakkers
DOI: 10.1242/dev.198937

Editor: Kenneth Poss

Review timeline

Original submission:	25 November 2020
Editorial decision:	28 December 2020
First revision received:	24 June 2021
Accepted:	4 August 2021

Original submission

First decision letter

MS ID#: DEVELOP/2020/198937

MS TITLE: Prrx1b restricts fibrosis and promotes Nrg1-dependent cardiomyocyte proliferation during zebrafish heart regeneration

AUTHORS: Dennis E.M. de Bakker, Esther Dronkers, Mara Bouwman, Marie Jose Goumans, Anke Smits, and Jeroen Bakkers

I have now received all the referees' reports on the above manuscript, and have reached a decision. The referees' comments are appended below, or you can access them online: please go to BenchPress and click on the 'Manuscripts with Decisions' queue in the Author Area.

As you will see, the referees express considerable interest in your work, but have some criticisms and recommend a revision of your manuscript before we can consider publication. If you are able to revise the manuscript along the lines suggested, which may involve further experiments, I will be happy receive a revised version of the manuscript. In particular please note that reviewers #1 and 2 feel that the data in the paper are insufficient for a conclusion that Prrx1b transcriptionally regulates nrg1 expression, and that further experimentation and/or a more cautious interpretation is warranted. Your revised paper will be re-reviewed by one or more of the original referees, and acceptance of your manuscript will depend on your addressing satisfactorily the reviewers' major concerns. Please also note that Development will normally permit only one round of major revision.

We are aware that you may be experiencing disruption to the normal running of your lab that make experimental revisions challenging. If it would be helpful, we encourage you to contact us to discuss your revision in greater detail. Please send us a point-by-point response indicating where you are able to address concerns raised (either experimentally or by changes to the text) and where you will not be able to do so within the normal timeframe of a revision. We will then provide further guidance. Please also note that we are happy to extend revision timeframes as necessary.

Please attend to all of the reviewers' comments and ensure that you clearly highlight all changes made in the revised manuscript. Please avoid using 'Tracked changes' in Word files as these are lost in PDF conversion. I should be grateful if you would also provide a point-by-point response detailing

© 2021. Published by The Company of Biologists under the terms of the Creative Commons Attribution License (https://creativecommons.org/licenses/by/4.0/).

how you have dealt with the points raised by the reviewers in the 'Response to Reviewers' box. If you do not agree with any of their criticisms or suggestions please explain clearly why this is so.

Reviewer 1

Advance summary and potential significance to field

The manuscript by de Bakker et al. demonstrates a heart regeneration defect in a zebrafish mutant for prrx1b. This is interesting since prrx1b is the first transcription factor found to be dispensable for embryonic development but required for efficient regeneration. The authors then go on to propose 2 models for how prrx1b expressing cells impact regeneration. First, that these cells are regulating cardiac scarring and ECM deposition. Second, that prrx1b regulates expression of the cardiac mitogen nrg1. We have significant concerns with the later model and more minor concerns with the first.

To support a role for Prrx1b+ cells regulating scar formation, de Bakker et al. use scRNA-seq to show that prrx1b mutants have a shift in populations of activated fibroblasts. Mutants demonstrated an increased proportion of fibroblasts expressing genes for collagen deposition and TGF-b activation. They then show that prrx1b mutants have increased collagen deposition using Sirius Red staining.

Comments for the author

Major comments
Model – Prrx1b regulates expression of cardiac mitogen nrg1
1. Injecting NRG1 to suppress prrx1b mutant phenotypes does not indicate that they work in the same pathway. At best it suggests that prrx1b is not required for NRG1 function. It does not place it upstream.

2. Nrg1 expression has never been detected by ISH. Gemberling et al. resorted to RNA scope to detect the lowly expressed transcripts. It does not appear that the cells marked by the ISH probe in de Bakker et al. (Fig. 5A) are the same as the perivascular cells described in Gemberling et al (Fig. 1 E,G). They are much more reminiscent of the periostin b expressing cells shown in Sanchez-Iranzo et al. (Fig. 4D). Are the authors sure that the ISH probe is detecting nrg1 transcripts and not something else?

3. If the authors wanted to formally demonstrate direct regulation of NRG1 by PRXX1 in cell culture they would have to ChIP the PRRX1 protein nearby to the NRG1 gene. This point is almost irrelevant because what happens in human cell lines is not informative to what happens in a zebrafish heart. It is likely technically prohibitive but a ChIP for Prrx1b from zebrafish hearts would be critical. The non-specific background demonstrated in the antibody (see #7 below) would make the interpretation of such results challenging.

4. The most robust Prrx1b expressing cells are quite distal to the wound.
Well within range to effect ECM deposition and scarring (model 1) but it is unlikely that a transmembrane protein like Nrg1 functions at this distance (model 2). NRG1 is activated locally by cleavage to activate across a typical synaptic cleft. Can the authors provide literature that NRG1 can function at this increased distance? If the DAPI was shown in Figure 2 (not necessary), it would be obvious that there are thousands of cells in between the proliferating muscle and Prrx1b+ cells.

5. “Prrx1b expression…initially localized at the epicardium and found more dispersed throughout the injury area at later stages” (last sentence in the second section).
In our view Prrx1b expression remains rather discretely localized except for a few rare cells. The localization is opposite from the injury surrounding the clot/scar. Why do the authors not mention this? This particular cluster of cells was identified in Sanchez-Iranzo et al. as periostin b expressing. However, the requirement of these cells for regeneration has not been specifically established. While tcf21+ cells are generally required (Wang et al.) and col1a2+ fibroblasts as well (Sanchez-Iranzo et al.), Prrx1b appears to mark a discrete subset of these two cell populations. From their localization and time course it strikes us as much more likely that Prrx1b+ cells are influencing

© 2021. Published by The Company of Biologists under the terms of the Creative Commons Attribution License (https://creativecommons.org/licenses/by/4.0/).

resolution of scar rather than nrg1 expression. Is nrg1 really expressed throughout regeneration and into 30 dpi (see Prrx1b expression in Figure 2F)? What is Prrx1b doing differently at these later time points if not? Due to the domain and time-course of expression we find it far more likely that Prrx1b+ cells are resolving scar and the effects on cardiomyocyte proliferation are likely indirect.

Minor comments
1. For the Sirius Red staining, it is possible that one of the mutant measurements is an outlier that when removed makes the difference insignificant (Fig. 4I). However, to our eye, it does appear that there will be a more subtle effect remaining. More N is needed to resolve this and buttress the collagen staining results.

2. The sections showing scar resolution in prrx1b mutants are difficult to interpret (Figure 1). They should be from the middle of the heart rather than the heart wall. How do you know that the injury is not on the other side of the heart and you are just missing it? Some of the injuries are not directly on the bottom of the heart. Please show more central sections as was done for cardiomyocyte proliferation in Figure 1E.

3. Figure 1B and 1D. What is relative scar size? Why is the scale different on each graph? If they are relative, they should both be on the same scale as a percentage, no? In our opinion, scar calculation at the later time point is probably unnecessary. Simply stating that 4 / 7 hearts retained the scar and showing an example as in Figure 1C should suffice.

4. It appears that at 90 dpi, 3 out of 7 mutants resolved their scar completely (Figure 1D). The phenotype may not be completely penetrant. Like in the embryo, prrx1a expression may compensate. We find it strange that a similar distribution is not observed in the proliferation data shown in Figure 1F. Can the authors comment?

5. IA is not the injured area but the very edge of the clot opposite the injured area (Figure 2).

6. The error bars in Supplemental Figure 4 are quite profound. How do the authors conclude that there is no difference between the conditions? Is this experiment sufficiently powered? It strikes us that the assay may simply be flawed, particularly the mCherry+ cell quantitation. Could it be that recombination of the Cre is not complete?

7. The antibody for Prrx1b has a lot of background. Ice crystals possibly?
While some of the signal is robust and as expected appears nuclear, most of the signal is diffuse and appears quite non-specific. While we think that does not render the reagent unusable, interpretations must be careful. It is not unbiased to ignore diffuse staining throughout the cytoplasm of trabecular cardiomyocytes and focus only on bright nuclei surrounding the wound. Could the authors at least show an ISH of the expression of the prrx1b transcript to justify focusing on some signals and not the others?

Reviewer 2

Advance summary and potential significance to field

In this report by de Bakker, Dronkers et al., the authors explore the role of the transcription factor prrx1 in scar-free regeneration in the zebrafish heart. They demonstrate that prrx1 is expressed in a subpopulation of tcf21+ EPDCs in injured hearts. Using sc-RNAseq they show heterogeneity in EPDC and fibroblast populations and that prrx1b-/- hearts contain more activated pro-fibrosis fibroblasts as opposed to quiescent fibroblasts in comparison to wildtype hearts.
In addition, the authors demonstrate that prrx1b is required for zebrafish cardiomyocyte proliferation and heart regeneration by subjecting prrx1b-/- fish to cardiac cryoinjury. This was rescued by ectopic Nrg1, a growth factor that has been previously shown to induce cardiomyocyte proliferation. Finally, the authors concluded that Prrx1 regulates Nrg1 expression in EPDCs based on the observation that PRRX1 KD using siRNAs in human foetal epicardial cells leads to a decrease in NRG1 mRNA and secreted NRG1-β1 protein. Overall, the work holds potential interest and utility among researchers in this field and would be a good fit for Development. Especially the

© 2021. Published by The Company of Biologists under the terms of the Creative Commons Attribution License (https://creativecommons.org/licenses/by/4.0/).

identification of different fibroblast populations after injury between scarring and regenerating hearts is of significant interest.
However, additional data and further considerations of existing data are needed to better support the authors' statements.

Comments for the author

Tcf21 is expressed in subpopulations of the epicardium and EPDCs. Therefore, it is not valid to claim that "all" EPDCs are marked in the Tg(tcf21:CreERT2) line.
This should be acknowledged in the paper.

For the lineage tracing with Tcf21creERT2 mcherry, at 1-14 dpi most prrx1 positive cells are also mcherry positive. At 30dpi, most prrx1 positive cells are mcherry negative. Is another cell type prrx1 positive at this stage? Or is mCherry staining too blurry to distinguish cells and if so, why would this be?

These results (Figure 2) are also too descriptive. Can the proportion of mcherry+ prrx1+ cells be quantified at the different locations and timepoints?

Prrx1 expression should be shown in the sc-RNAseq data. In addition, it would be of interest to see which epicardial subpopulation expresses Prrx1: tbx18+? wt1+?
This can be shown using ISH or in the sc-RNAseq data.

In Figure 4F and -G ISH, tgfb1a and col11a1b seem to be much higher expressed in the whole heart of prrx1b-/- fish compared to wildtype. Is this a technical problem or is the effect visible in the whole ventricle?

The siRNA experiment is not sufficient to conclude that Prrx1 regulates Nrg1 and therefore this should at least be toned down in the text. Perhaps investigate if the same effect is observed in a different cell type. Does nrg1 specifically overlap with prrx1 expression in fish? Is there an effect of PRRX1 KD on epicardial spindle cell formation and cell numbers?

siRNA experiment in Figure 5H: a negative siRNA (CTRL siRNA) is included but nontransfected cells should also be shown. KD should also be confirmed by western blot.

One of the main findings in this paper is the heterogeneity of fibroblast populations. It would be of interest to at least link this to the rest of the findings by looking at tgfb1a and col11a1b expression in the rescued fish hearts or in vitro.

Statistical analysis in Figure 5C and Figure S4 is not described. In the methods section, the authors mention that unless stated otherwise, all statistical testing was performed by unpaired T-tests but T-tests are not applicable here.
Minor points:
- Figure S3 should replace Figure 2
- Show individual values in Figure 5F and -H
- Mention how many hearts were analysed in Figure 3 and Figure 4 ISH

<u>Reviewer 3</u>

Advance summary and potential significance to field

The study presents a thorough characterization of the expression of the transcription factor paired related homeobox 1 (Prrx1) in epicardial-derived cells (EPDCs) during zebrafish cardiac regeneration and its requirement in fibrotic scar regression. The results obtained by transcriptional analysis of wild-type and Prrx1-mutant hearts show that loss of the Prrx1-gene leads to an overrepresentation of pro-fibrotic fibroblasts upon cryoinjury.
Finally, the authors use both zebrafish in vivo and human in vitro models to demonstrate that Prrx1-expression in EPDCs induces cardiomyocyte proliferation by promoting the expression of Nrg1. Overall, this is a well-written manuscript in which both the experimental assessment and the

© 2021. Published by The Company of Biologists under the terms of the Creative Commons Attribution License (https://creativecommons.org/licenses/by/4.0/).

conclusions drawn upon soundly demonstrate the importance of Prrx1b in zebrafish heart regeneration and complement the existing literature on the role of EPDCs in the restoration of cardiac tissue.

Comments for the author

Minor comments:
• Results section.
o Please address the specificity of Prrx1-expression in EPDCS compared to CMs (as depicted in Supp. Fig. 2) in the results section.
o Is there a particular reason why Prrx1-expression was not addressed at the mRNA level?
• Methods section:
o Please correct: " The next day, the hearts were washed 3x 10 minutes in 4% sucrose phosphate buffer, after which they were incubated at RT for at least 5h in 30% sucrose phosphate buffer until the hearts sank".
o According to the description of quantification analysis "surfaces inside the injury were selected manually to create a subset of the total surface". Does "total surface area" refers to the total injury surface area?
If so, what was the criterium for the three sections used for the analysis?
o Please state if quantifications were performed blinded.
• Discussion section:
o Please consider the following study when addressing the outcome and impact of the results: Hortells et al., 2020. "A specialized population of Periostin-expressing cardiac fibroblasts contributes to postnatal cardiomyocyte maturation and innervation" PNAS, 117(35):21469-21479. doi: 10.1073/pnas.2009119117.

First revision

Author response to reviewers' comments

Prrx1b restricts fibrosis and promotes Nrg1-dependent cardiomyocyte proliferation during zebrafish heart regeneration

Addressing reviewers' comments

We would like to thank all reviewers for their constructive feedback and helpful suggestions to improve the manuscript. We have addressed all comments point-by-point below and our responses are marked in blue text. Changes in the manuscript are also marked in blue.

Reviewer 1

Advance Summary and Potential Significance to Field:

The manuscript by de Bakker et al. demonstrates a heart regeneration defect in a zebrafish mutant for prrx1b. This is interesting since prrx1b is the first transcription factor found to be dispensable for embryonic development but required for efficient regeneration. The authors then go on to propose 2 models for how prrx1b expressing cells impact regeneration. First, that these cells are regulating cardiac scarring and ECM deposition. Second, that prrx1b regulates expression of the cardiac mitogen nrg1. We have significant concerns with the later model and more minor concerns with the first.

To support a role for Prrx1b+ cells regulating scar formation, de Bakker et al. use scRNA-seq to show that prrx1b mutants have a shift in populations of activated fibroblasts. Mutants demonstrated an increased proportion of fibroblasts expressing genes for collagen deposition and TGF-b activation.
They then show that prrx1b mutants have increased collagen deposition using Sirius Red staining.

© 2021. Published by The Company of Biologists under the terms of the Creative Commons Attribution License (https://creativecommons.org/licenses/by/4.0/).

Major comments

Model – Prrx1b regulates expression of cardiac mitogen nrg1
1. Injecting NRG1 to suppress prrx1b mutant phenotypes does not indicate that they work in the same pathway. At best it suggests that prrx1b is not required for NRG1 function. It does not place it upstream.

Authors' response:
We agree with the reviewer that rescuing the cardiomyocyte proliferation defect in the prrx1b mutant by injecting NRG1 by itself is not sufficient to draw conclusions about the relation between Prrx1b and Nrg1. Besides the rescue experiment we provide additional experimental data that support our model in which Prrx1 acts upstream of Nrg1:

- Prrx1 and nrg1 are co-expressed in the sub-epicardial region of cryoinjured hearts. We now performed RNAscope analysis for nrg1 (see comment#2 below) expression, which was combined with the Prrx1 antibody. Form this we conclude that *nrg1* and Prrx1 are co- expressed in BZ epicardial cells. These new results have now been included in Figure 5 and are described on page 9 of the revised manuscript.
- nrg1 expression is strongly reduced in prrx1b mutants. In the original manuscript we performed classical ISH on sections of wild type and prrx1b mutant hearts and found a clear reduction of the signal in the mutant hearts. We now have repeated this experiment using the RNAscope technique to detect nrg1 expression and come to very similar conclusions. These new data are shown in Figure 5 and described on page 9.
- NRG1 and PRRX1 are both expressed in human fetal epicardial derived cells and RNAi knock-down of PRRX1 results in reduced NRG1 expression and reduced NRG1 protein secretion.

Together these results indicate that the level of Nrg1 expression depends on Prrx1. Whether there is direct regulation of Nrg1 by Prrx1 or not needs further investigations. We have tried to addsress this by performing chromatin immunoprecipitation (ChIP) experiments with the Prrx1 antibody but were unsuccessful (also see comment#3 below).

2. Nrg1 expression has never been detected by ISH. Gemberling et al. Resorted to RNA scope to detect the lowly expressed transcripts. It does not appear that the cells marked by the ISH probe in de Bakker et al. (Fig. 5A) are the same as the perivascular cells described in Gemberling et al (Fig. 1E,G). They are much more reminiscent of the periostin b expressing cells shown in Sanchez-Iranzo et al. (Fig. 4D). Are the authors sure that the ISH probe is detecting nrg1 transcripts and not something else?

Authors' response:
To address this we now have performed RNAscope analysis for nrg1 using the same nrg1 probe as was previously published by Gemberling et al. Using this approach, we observed clear nrg1 expression in the sub-epicardial region of cryoinjured hearts. Whether these nrg1-expressing cells include perivascular cells we could not address since we did not have a marker for these cells. We did however co-stain with the Prrx1 antibody and observed co-expression of nrg1 and Prrx1 (indicating that these are epicardial-derived). We noticed abundant nrg1 expression in the sub-epicardial regions near the border zone (here referred to as the border zone epicardium region), which is also the site that is indicated in Fig.1G in the Gemberling manuscript as a region with robust nrg1 expression. This result demonstrates that we indeed detect nrg1 transcripts and not something else.
We also repeated the experiment in which we addressed nrg1 expression in prrx1b mutant hearts. Similar to what we reported for the conventional ISH in the original manuscript, we also observed a reduction in nrg1 expression in the prrx1b mutant heart using the RNAscope approach. We also quantified the amount of nrg1 signal in the border zone epicardium region and found a significant reduction in nrg1 in prrx1b mutants. This further supports our model that nrg1 expression is regulated by Prrx1. These RNAscope results are shown in Figure 5 and described on page 9.

3. If the authors wanted to formally demonstrate direct regulation of NRG1 by PRXX1 in cell culture they would have to ChIP the PRRX1 protein nearby to the NRG1 gene. This point is almost

© 2021. Published by The Company of Biologists under the terms of the Creative Commons Attribution License (https://creativecommons.org/licenses/by/4.0/).

irrelevant because what happens in human cell lines is not informative to what happens in a zebrafish heart. It is likely technically prohibitive but a ChIP for Prrx1b from zebrafish hearts would be critical. The non- specific background demonstrated in the antibody (see #7 below) would make the interpretation of such results challenging.

Authors' response:
We agree that a direct regulation of Nrg1 by Prrx1 would require a ChIP experiment. We performed ChIP experiments on human fetal epicardial spindle cells using the Prrx1 antibody. However, we were unable to enrich for sequences with Prrx1 bindings motifs upstream of coding regions known to be regulated by Prrx1 in mammals. This is likely due to the quality of the Prrx1 antibody, which might not be ChIP grade. For example, we were unable to get good results using a SOX9 regulatory region as a positive control (Reichert et al (2013) Genes Dev.; PMID: 23355395). In this published study, a flag tag and anti-flag antibody was used for the ChIP experiments. We therefore cannot conclude whether Nrg1 regulation by Prrx1 is direct or indirect which we also stated in the discussion on page 12:
"*Both the in vitro experiments in human fetal EPDCs and the in vivo experiments in zebrafish demonstrate that Nrg1 expression depends on Prrx1. Whether this is through binding of Prrx1 to regulatory sequences in the Nrg1 locus or whether is through a more indirect mechanism needs to be further investigated for example by chromatin immunoprecipitation (ChIP) experiments.*"

4. The most robust Prrx1b expressing cells are quite distal to the wound. Well within range to effect ECM deposition and scarring (model 1) but it is unlikely that a transmembrane protein like Nrg1 functions at this distance (model 2). NRG1 is activated locally by cleavage to activate across a typical synaptic cleft. Can the authors provide literature that NRG1 can function at this increased distance? If the DAPI was shown in Figure 2 (not necessary), it would be obvious that there are thousands of cells in between the proliferating muscle and Prrx1b+ cells.

Authors' response:
Nrg1 is expressed as a proNrg1 protein that has a transmembrane domain. There are several reports in which it was demonstrated that proNrg1 is cleaved, which releases it into the extracellular space (pmids: 11083924, 7642587). This is consistent with its suggested paracrine role during development.

In the developing heart Nrg1 is expressed in the endocardial cushions. Expression of Nrg1 in the endocardial cells is required for trabeculation of the myocardium where its receptor ErbB2 is expressed (pmids: 7477375, 16481353, 20978078). Consistent with this, we and others have injected recombinant NRG1 protein intraperitoneal and observed effects on cardiomyocyte proliferation.
Besides expression of nrg1 in cells distal to the wound we observed robust nrg1 and prrx1b expression in the sub-epicardial region directly adjacent to the border zone, where most of the injury- induced cardiomyocyte proliferation occurs. In these border zone epicardial regions secreted Nrg1 does not need to diffuse very far to reach the border zone cardiomyocytes.

5. "Prrx1b expression…initially localized at the epicardium and found more dispersed throughout the injury area at later stages" (last sentence in the second section).
In our view Prrx1b expression remains rather discretely localized except for a few rare cells. The localization is opposite from the injury surrounding the clot/scar. Why do the authors not mention this? This particular cluster of cells was identified in Sanchez-Iranzo et al. as periostin b expressing. However, the requirement of these cells for regeneration has not been specifically established. While tcf21+ cells are generally required (Wang et al.) and col1a2+ fibroblasts as well (Sanchez-Iranzo et al.), Prrx1b appears to mark a discrete subset of these two cell populations. From their localization and time course it strikes us as much more likely that Prrx1b+ cells are influencing resolution of scar rather than nrg1 expression. Is nrg1 really expressed throughout regeneration and into 30 dpi (see Prrx1b expression in Figure 2F)? What is Prrx1b doing differently at these later time points if not? Due to the domain and time-course of expression we find it far more likely that Prrx1b+ cells are resolving scar and the effects on cardiomyocyte proliferation are likely indirect.

Authors' response:
We have reanalyzed the expression of Prrx1 in this revised version also based on the comments by reviewer 2. This includes a more detailed analysis of the localization of Prrx1 expressing cells and

© 2021. Published by The Company of Biologists under the terms of the Creative Commons Attribution License (https://creativecommons.org/licenses/by/4.0/).

quantification of the results which are presented in the new Figure 2. In this analysis we quantified the number of Prrx1+/tcf21:mCherry+ cells in four regions: remote epicardium, border zone epicardium, injury epicardium and in injury. Interestingly we observed that the accumulation of Prrx1+ cells in the border zone epicardium is most apparent at 3 and 7 dpi, which coincides with the time points when cardiomyocyte proliferation is peaking. In the injury area, we found that Prrx1+ cells appear at 7 dpi and are still present at 30 dpi. The latter may be related to scar resolution in the injury area. Whether the effects of Prrx1 on cardiomyocyte proliferation and scar resolution are independent of each other or are related is an interesting question but will require extensive new investigations and the generation of several new transgenic lines.

Minor comments

1. For the Sirius Red staining, it is possible that one of the mutant measurements is an outlier that when removed makes the difference insignificant (Fig. 4I). However, to our eye, it does appear that there will be a more subtle effect remaining. More N is needed to resolve this and buttress the collagen staining results.

Authors' response:
The conclusion of the experiment is not affected by the possible outlier in the mutant data. When we remove this data point the difference between the wild type and mutant scar size remains significant (p value is even smaller when the data point is removed). Please see Figure R1.1 below.

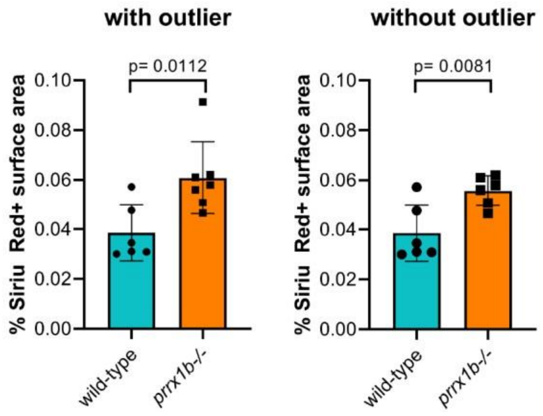

Figure R1.1: Quantification of Sirius Red staining in wild-type and *prrx1b-/-* hearts including the outlier as in Fig.4I of the manuscript (left) and excluding the outlier (right). The difference between wild-type and *prrx1b-/-* hearts remains significant.

2. The sections showing scar resolution in prrx1b mutants are difficult to interpret (Figure 1). They should be from the middle of the heart rather than the heart wall. How do you know that the injury is not on the other side of the heart and you are just missing it? Some of the injuries are not directly on the bottom of the heart. Please show more central sections as was done for cardiomyocyte proliferation in Figure 1E.

Authors' response:
When we analyze the scar size, we make sure that we section and stain the entire heart so that we will not miss any remaining scar tissue. What we show in Figure 1 are the sections with the largest scar surface, which not necessarily need to be in the central part of the heart.

3. Figure 1B and 1D. What is relative scar size? Why is the scale different on each graph? If they are relative, they should both be on the same scale as a percentage, no? In our opinion, scar calculation at the later time point is probably unnecessary. Simply stating that 4 / 7 hearts retained the scar and showing an example as in Figure 1C should suffice.

Authors' response:
We apologize for making a mistake in the description of the y-axis. Instead of 'relative scar size' these should read 'Average scar size in μm^2' and have been adjusted.

© 2021. Published by The Company of Biologists under the terms of the Creative Commons Attribution License (https://creativecommons.org/licenses/by/4.0/).

4. It appears that at 90 dpi, 3 out of 7 mutants resolved their scar completely (Figure 1D). The phenotype may not be completely penetrant. Like in the embryo, prrx1a expression may compensate. We find it strange that a similar distribution is not observed in the proliferation data shown in Figure 1F. Can the authors comment?

Authors' response:
We think that this observation could be related to a dual role of Prrx1 in regulating cardiomyocyte proliferation and scar resolution as we explained above. The penetrance of the two observed phenotypes (CM proliferation and scar resolution) might therefore differ from each other in the *prrx1b* mutants, since these processes (while likely inter-dependent to a certain extent) might be relatively more or less dependent on *prrx1b*.

5. IA is not the injured area but the very edge of the clot opposite the injured area (Figure 2).

Authors' response:
As mentioned in our response to major comment 5, we have redefined the regions and improved Figure 2. It now includes a distinction between cells of the injury epicardium, and cells within the injury.

6. The error bars in Supplemental Figure 4 are quite profound. How do the authors conclude that there is no difference between the conditions? Is this experiment sufficiently powered? It strikes us that the assay may simply be flawed, particularly the mCherry+ cell quantitation. Could it be that recombination of the Cre is not complete?

Authors' response:
We agree that the error bars are profound, which may be due to the experimental system in which Cre-recombination varies across samples. We have now analyzed more samples to increase the number of data points but this did not make any differences in the outcome and conclusions. We have included the extra data in Supplemental figure 4. In addition, we address the raised issue in the description of the presented results (page 7): "*Taken together, we did not find evidence suggesting a profound role for Prrx1 in epicardial proliferation or EMT. Although the incomplete labelling of tcf21 + cells resulted in substantial variation in the collected data, potentially masking a subtle difference between wild-type and prrx1b-/- hearts, we conclude that Prrx1 is largely dispensable for proliferation and invasion into the injury area of epicardial and epicardial derived cells.*"

7. The antibody for Prrx1b has a lot of background. Ice crystals possibly? While some of the signal is robust and as expected appears nuclear, most of the signal is diffuse and appears quite non-specific. While we think that does not render the reagent unusable, interpretations must be careful. It is not unbiased to ignore diffuse staining throughout the cytoplasm of trabecular cardiomyocytes and focus only on bright nuclei surrounding the wound. Could the authors at least show an ISH of the expression of the prrx1b transcript to justify focusing on some signals and not the others?

Authors' response:
We performed ISH for *prrx1b* on injured hearts, but the results were not very conclusive as the signal was very weak near detection levels. A representative ISH image is now included in Figure S2A. The very low level of *prrx1b* mRNA was also observed in the scRNAseq data (see Fig. S6) and in our previous Tomo-seq approach in which we only detected two reads for prrx1b in the whole heart see our tomo-seq database: http://zebrafish.genomes.nl/tomoseq/Kruse2015/gene.pl)
Because of the low detection by ISH we reverted to using the anti-Prrx1 antibody instead. To address the specificity of the anti-Prrx1 antibody we have performed immunohistochemistry on serial sections of the same heart using either only the secondary antibody or using the primary anti-Prrx1 antibody together with the secondary antibody (see Figure R1.2 below). As can be appreciated in the secondary only image, there is autofluorescence of the cardiomyocytes. This diffuse autofluorescence of the cardiomyocytes is what we always observe in our immunohistochemistry pictures and is independent of the primary antibody used. The panel with the anti-Prrx1 antibody shows that the specific nuclear Prrx1 signal is only observed in the epicardium, which we focused on in the rest of our study. In addition, the anti-Prrx1 antibody was used on sections of *prrx1b-/-* hearts (Figure S2C), in which the nuclear and epicardial signal is

© 2021. Published by The Company of Biologists under the terms of the Creative Commons Attribution License (https://creativecommons.org/licenses/by/4.0/).

almost completely lost. We realized that in Figure S2 of the original manuscript the signal intensity of the *prrx1b-/-* hearts was increased a lot to visualize some weak staining in the epicardium and that this also increased the diffuse background signal in the myocardium. In the revised version of Figure S2 we now included pictures of both wild type and *prrx1b* mutant hearts with identical settings to allow a better comparison. These pictures show a dramatic reduction of the specific signal in *prrx1b-/-* while the diffuse background (autofluorescence) in wild type and *prrx1b-/-* is comparable. We also included now the single channel pictures that make the interpretation of the data easier.

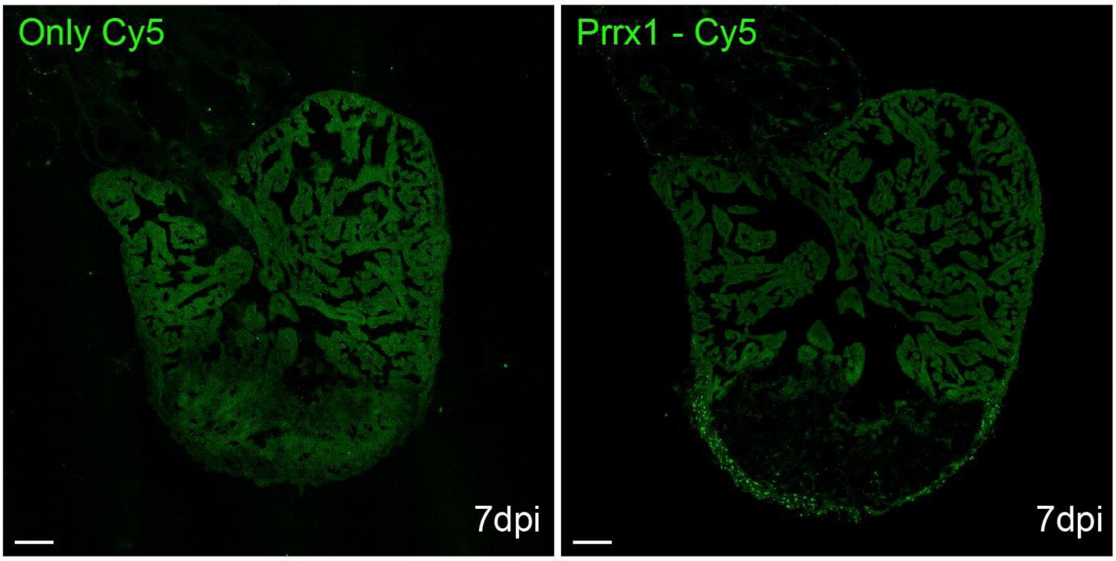

Figure R1.2. Immunofluorescent staining on 7dpi wild-type hearts using only secondary antibody Cy5 (left) and both primary anti-Prrx1 antibody and secondary antibody Cy5 (right). Autofluorescence of the myocardium is similar in both conditions. Scalebars represent 100µm.

Reviewer 2

Advance Summary and Potential Significance to Field:

In this report by de Bakker, Dronkers et al., the authors explore the role of the transcription factor prrx1 in scar-free regeneration in the zebrafish heart. They demonstrate that prrx1 is expressed in a subpopulation of tcf21+ EPDCs in injured hearts. Using sc-RNAseq they show heterogeneity in EPDC and fibroblast populations and that prrx1b-/- hearts contain more activated pro-fibrosis fibroblasts as opposed to quiescent fibroblasts in comparison to wildtype hearts. In addition, the authors demonstrate that prrx1b is required for zebrafish cardiomyocyte proliferation and heart regeneration by subjecting prrx1b-/- fish to cardiac cryoinjury. This was rescued by ectopic Nrg1, a growth factor that has been previously shown to induce cardiomyocyte proliferation. Finally, the authors concluded that Prrx1 regulates Nrg1 expression in EPDCs based on the observation that PRRX1 KD using siRNAs in human foetal epicardial cells leads to a decrease in NRG1 mRNA and secreted NRG1-ß1 protein. Overall, the work holds potential interest and utility among researchers in this field and would be a good fit for Development. Especially the identification of different fibroblast populations after injury between scarring and regenerating hearts is of significant interest. However, additional data and further considerations of existing data are needed to better support the authors' statements.

Major comments

1. Tcf21 is expressed in subpopulations of the epicardium and EPDCs. Therefore, it is not valid to claim that "all" EPDCs are marked in the Tg(tcf21:CreERT2) line. This should be acknowledged in the paper.

Authors' response:
We agree with the reviewer that tcf21 is only expressed in a subpopulation of EPDCs and we have

© 2021. Published by The Company of Biologists under the terms of the Creative Commons Attribution License (https://creativecommons.org/licenses/by/4.0/).

now clearly stated this in the text on page 5:
"To validate this we used the Tg(tcf21:CreERT2) line which marks a subset of EPDCs when crossed with the ubiquitous reporter Tg(ubi:loxP-EGFP-loxP-mCherry) and recombined during embryonic heart development (Kikuchi et al., 2011)."
In addition, we have now also analyzed the expression of Prrx1 in other subpopulations of epicardial cells/EPDCs, which are identified by expression of Tbx18 or Wt1 (see point 3 below).

2. For the lineage tracing with Tcf21creERT2 mcherry, at 1-14 dpi most prrx1 positive cells are also mcherry positive. At 30dpi, most prrx1 positive cells are mcherry negative. Is another cell type prrx1 positive at this stage? Or is mCherry staining too blurry to distinguish cells and if so, why would this be? These results (Figure 2) are also too descriptive. Can the proportion of mcherry+ prrx1+ cells be quantified at the different locations and timepoints?

Authors' response:
Based on the suggestions by the reviewer we have reanalyzed the localization of Prrx1+ cells in the cryoinjured heart and we quantified the Prrx1+/mCherry+ cells at different locations and time points. The results are presented in the revised Figure 2. In this analysis we quantified the number of Prrx1+/mCherry+ cells in four regions: remote epicardium, border zone epicardium, injury epicardium and in injury. Interestingly we observed that the accumulation of Prrx1+ cells in the border zone epicardium (sub-epicardial region near the border zone) is most apparent at 3 and 7 dpi, which coincides with the time points when cardiomyocyte proliferation is high. In the injury area, we found that Prrx1+ cell appear at 7 dpi and are still present at 30 dpi. The latter may be related to scar resolution in the injury area.
The quantification in Figure 2H shows that at 30 dpi there are still Prrx1+/mCherry+ cells (~30% of all Prrx1+ cells) but the total number of Prrx1+/mCherry+ cells has decreased dramatically to <10 in the different regions. Considering the quantification, we agree with the reviewer that at 30dpi we observe many Prrx1+/mCherry- cells. This might be due to incomplete recombination induced by the tamoxifen, leaving a sub-set of Tcf21+ cells unlabeled. In addition, it could be that the observed Prrx1+/mCherry- cells represent tbx18+ or wt1b+ EPDC populations, which we co-stained following the next comment of the reviewer (#3) (and is now included in the manuscript as Figure S3).

3. Prrx1 expression should be shown in the sc-RNAseq data. In addition, it would be of interest to see which epicardial subpopulation expresses Prrx1: tbx18+? wt1+? This can be shown using ISH or in the sc-RNAseq data.

Authors' response:
To address which epicardial subpopulation expresses Prrx1 we could not use the scRNAseq data as the number of detected prrx1b reads was too low (now included as Fig.S6). Instead, we have performed immunohistochemistry with the anti-Prrx1 antibody on cryoinjured hearts of *Tg(tbx18:myr- eGFP)* and *Tg(wt1b:H2B-Dendra2)* fish. These new results are now presented in Supplementary Figure S3 and described on page 6. From this we conclude that Prrx1 is expressed across at least 3 different epicardial subpopulations.

4. In Figure 4F and -G ISH, tgfb1a and col11a1b seem to be much higher expressed in the whole heart of prrx1b-/- fish compared to wildtype. Is this a technical problem or is the effect visible in the whole ventricle?

Authors' response:
We agree with the reviewer that there is a small visible effect in tgfb1a and col11a1b expression the whole heart of prrx1b-/- fish. Although we cannot exclude small differences between wild-type and mutant in the myocardium in terms of tgfb1 and col11a1b expression, the signal appears to be ubiquitous throughout the heart, and therefore we doubt the specificity of the signal and think it might actually represent background signal.

5. The siRNA experiment is not sufficient to conclude that Prrx1 regulates Nrg1 and therefore this should at least be toned down in the text. Perhaps investigate if the same effect is observed in a different cell type. Does nrg1 specifically overlap with prrx1 expression in fish? Is there an effect of PRRX1 KD on epicardial spindle cell formation and cell numbers?

© 2021. Published by The Company of Biologists under the terms of the Creative Commons Attribution License (https://creativecommons.org/licenses/by/4.0/).

Authors' response:
We agree with the reviewer that the siRNA experiment is not sufficient to conclude that Prrx1 regulates NRG1 expression. Therefore we have changed this into: "*From these results we conclude that in EPDCs after EMT induction, PRRX1 and NRG1 are co-expressed and that PRRX1 is required for efficient NRG1 expression.*" We did not address this effect in another cell type as we did not have a cell line available in which PRRX1 and NRG1 are coexpressed. We did perform extra experiments in the zebrafish that relate to this:

- Prrx1 and nrg1 are co-expressed in the sub-epicardial region of cryoinjured hearts. We now performed RNAscope analysis for nrg1 (see comment#2 below) expression, which was combined with the Prrx1 antibody. Form this we conclude that *nrg1* and Prrx1 are co- expressed in BZ epicardial cells. These new results have now been included in Figure 5 and are described on page 9 of the revised manuscript.
- nrg1 expression is strongly reduced in prrx1b mutants. In the original manuscript we performed classical ISH on sections of wild type and prrx1b mutant hearts and found a clear reduction of the signal in the mutant hearts. We now have repeated this experiment using the RNAscope technique to detect nrg1 expression and come to very similar conclusions. These new data are shown in Figure 5 and described on page 9.

Together these results indicate that the level of Nrg1 expression depends on Prrx1. Whether there is direct regulation of Nrg1 by Prrx1 or not requires further investigation. We have tried to address this by performing chromatin immunoprecipitation (ChIP) experiments with the Prrx1 antibody but were unsuccessful (also see response to comment#3 of reviewer1).
Questioning if PRRX1 KD has an effect on spindle cell differentiation or cell numbers is valid as it may influence NRG-1 expression. However, before the start of the siRNA experiment the cells were already fully differentiated towards mesenchymal spindles so we anticipate that any effect of PRRX1 KD on differentiation cannot have affected NRG-1 expression. To confirm this, we tested the effect of PRRX1 KD on the expression of EMT genes SNAIL and SLUG in vitro but we could not establish an effect (see additional figure R2.1). Regarding the cell numbers, we have never observed an effect of PRRX1 KD on cell numbers. Additional figure R2.2 shows a representative example of cell counts at the end of the PRRX1 siRNA experiment which does not indicate an effect of PRRX1 KD on cell numbers.

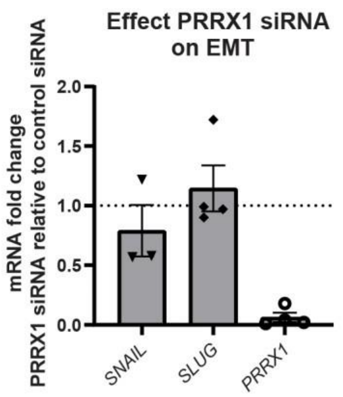

Figure R2.1: mRNA fold change of SNAIL, SLUG and PRRX1 expression after PRRX1 siRNA compared to control siRNA, showing no significant effect of PRRX1 KD on the expression of EMT genes SNAIL and SLUG.

© 2021. Published by The Company of Biologists under the terms of the Creative Commons Attribution License (https://creativecommons.org/licenses/by/4.0/).

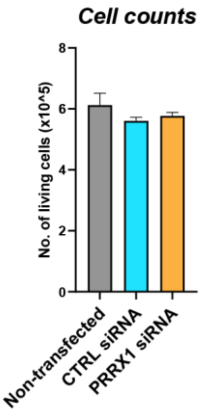

Figure R2.2: Cell counts after PRRX1 siRNA experiment showing no effect of PRRX1 KD on cell numbers.

6. siRNA experiment in Figure 5H: a negative siRNA (CTRL siRNA) is included but nontransfected cells should also be shown. KD should also be confirmed by western blot.

Authors' response:
We agree with the reviewer that the non-transfected cells is a valuable addition to the graph and we have adapted the figure (now labelled as Figure 6). Furthermore, we confirmed the ability of the siRNA to KD PRRX1 expression by western blot as seen in figure 6E.

7. One of the main findings in this paper is the heterogeneity of fibroblast populations. It would be of interest to at least link this to the rest of the findings by looking at tgfb1a and col11a1b expression in the rescued fish hearts or in vitro.

Authors' response:
We agree with the reviewer that it would be of interest to further investigate the heterogeneity of the fibroblasts and the role of Prrx1 herein. We tried to address this by looking at tgfb1a and col11a1b expression in the NRG1 rescued hearts as suggested by the reviewer, but this did not give consistent results possibly due to the experimental setup with injection of recombinant NRG1. More extensive experiments including the generation of additional transgenic lines would be required to investigate the link between the different fibroblast states identified by the scRNAseq experiment and the role of Prrx1 in regulating cardiomyocyte proliferation and scar resolution. We feel that this is outside the scope of this manuscript.

8. Statistical analysis in Figure 5C and Figure S4 is not described. In the methods section, the authors mention that unless stated otherwise, all statistical testing was performed by unpaired T-tests but T tests are not applicable here.

Authors' response:
We apologize for this mistake in our statistical analyses. We have now applied the suitable test to these data, a one-way Anova followed by a multiple comparisons analysis using the Tukey's test. We have adjusted the graph and p values accordingly in the new figure 5 and included the description in our methods.

Minor points:

•Figure S3 should replace Figure 2

Authors' response:
We have replaced Figure 2 by Figure S3 and updated this new Figure 2 with additional zoom ins and quantifications.

•Show individual values in Figure 5F and –H

Authors' response:
We have incorporated the individual values for these graphs in Figure 5F,5H and 5I, which in the

© 2021. Published by The Company of Biologists under the terms of the Creative Commons Attribution License (https://creativecommons.org/licenses/by/4.0/).

revised manuscript correspond to Figure 6C, 6F and 6G.

•Mention how many hearts were analysed in Figure 3 and Figure 4 ISH

Authors' response:
In the description of both Figure 3 and Figure 4 we mention that 3 hearts are analyzed per condition.

Reviewer 3

Advance Summary and Potential Significance to Field:

The study presents a thorough characterization of the expression of the transcription factor paired related homeobox 1 (Prrx1) in epicardial-derived cells (EPDCs) during zebrafish cardiac regeneration and its requirement in fibrotic scar regression. The results obtained by transcriptional analysis of wild-type and Prrx1-mutant hearts show that loss of the Prrx1-gene leads to an overrepresentation of pro- fibrotic fibroblasts upon cryoinjury. Finally, the authors use both zebrafish in vivo and human in vitro models to demonstrate that Prrx1-expression in EPDCs induces cardiomyocyte proliferation by promoting the expression of Nrg1. Overall, this is a well-written manuscript in which both the experimental assessment and the conclusions drawn upon soundly demonstrate the importance of Prrx1b in zebrafish heart regeneration and complement the existing literature on the role of EPDCs in the restoration of cardiac tissue.

Reviewer 3 Comments for the Author:

Minor comments:

•*Results section.*
Please address the specificity of Prrx1-expression in EPDCS compared to CMs (as depicted in Supp. Fig. 2) in the results section.

Authors' response:
To address the specificity of the anti-Prrx1 antibody we have performed immunohistochemistry on serial sections of the same heart using either only the secondary antibody or using the primary anti-Prrx1 antibody together with the secondary antibody (see Figure R3.1 below). As can be appreciated in the secondary only image, there is autofluorescence of the cardiomyocytes. This diffuse autofluorescence of the cardiomyocytes is what we always observe in our immunohistochemistry picture and is independent of the primary antibody used. The panel with the anti-Prrx1 antibody shows that the specific nuclear Prrx1 signal is only observed in the epicardium, which we focused on in the rest of our study. In addition, the anti-Prrx1 antibody was used on sections of *prrx1b-/-* hearts (Figure S2C), in which the nuclear and epicardial signal is almost completely lost. We realized that in Figure S2 of the original manuscript the signal intensity of the *prrx1b-/-* hearts was increased a lot to visualize some weak staining in the epicardium and that this also increased the diffuse background signal in the myocardium. In the revised version of Figure S2 we now included pictures of both wild type and *prrx1b* mutant hearts with identical settings to allow a better comparison. These pictures show a dramatic reduction of the specific signal in *prrx1b-/-* while the diffuse background (autofluorescence) in wild type and *prrx1b-/-* is comparable. We also included now the single channel pictures the make the interpretation of the data easier.

© 2021. Published by The Company of Biologists under the terms of the Creative Commons Attribution License (https://creativecommons.org/licenses/by/4.0/).

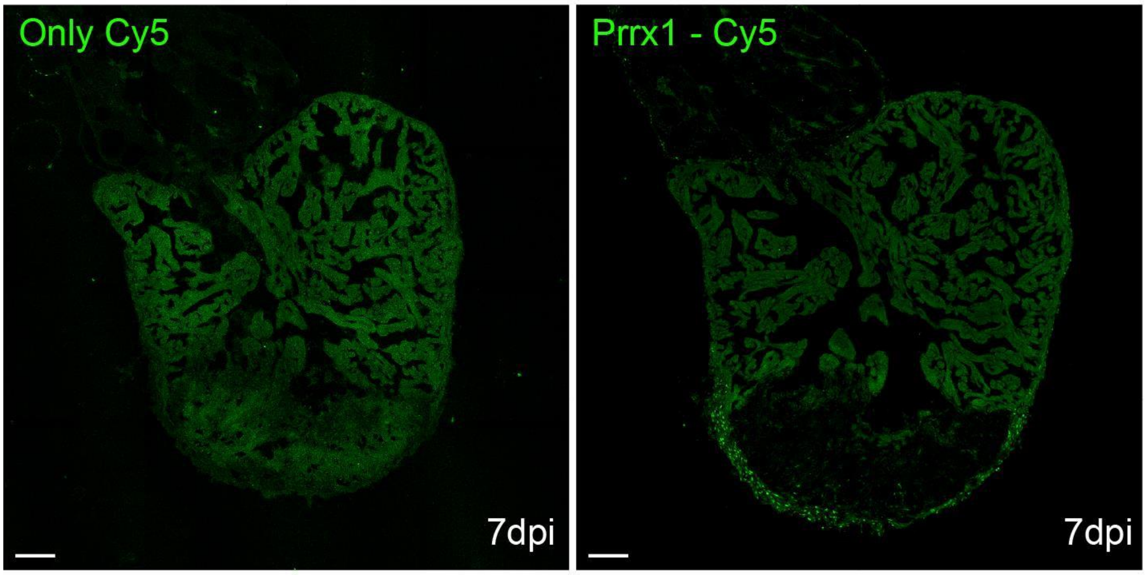

Figure R3.1. Immunofluorescent staining on 7dpi wild-type hearts using only secondary antibody Cy5 (left) and both primary anti-Prrx1 antibody and secondary antibody Cy5 (right). Autofluorescence of the myocardium is similar in both conditions. Scalebars represent 100µm.

Is there a particular reason why Prrx1-expression was not addressed at the mRNA level?
Authors' response:
We had not included the *prrx1b in situ* hybridization pictures in the original manuscript as their results were not very conclusive. We only detected a very weak signal that was near the detection level for the ISH. A representative ISH image is now included in Figure S2A. The very low level of *prrx1b* mRNA was also observed in the scRNAseq data (see Fig. S6) and in our previous Tomo-seq approach in which we only detected two reads for prrx1b in the whole heart see our Tomo-seq database: http://zebrafish.genomes.nl/tomoseq/Kruse2015/gene.pl). Because of the detection issues of the mRNA we used the anti-Prrx1 antibody instead to localize the protein, which showed a much better signal.

Methods section:

o Please correct: " The next day, the hearts were washed 3x 10 minutes in 4% sucrose phosphate buffer, after which they were incubated at RT for at least 5h in 30% sucrose phosphate buffer until the hearts sank".

Authors' response:
We apologize for this mistake in our methods and have now corrected it.

According to the description of quantification analysis "surfaces inside the injury were selected manually to create a subset of the total surface". Does "total surface area" refers to the total injury surface area? If so, what was the criterium for the three sections used for the analysis?

Authors' response:
We realize that our description of quantifications was unclear and have adjusted this in our revised methods. The total surface area referred to the total tcf21:mCherry+ surface of the whole injury area. We have now rephrased this in our methods. The whole injury area is defined as all tcf21:mCherry+ surface of the injured area, including 100um of border zone. As these surfaces include tcf21:mCherry+ cells in the outer layers of injury epicardium as well as surfaces of cells that have invaded into the injury, we manually subsetted the surfaces within the injury and present this as a percentage of the surface of the whole injury area as a measure of invasion. In general, we choose three heart sections with the largest injury for quantification analyses, which was also the case for these quantifications.

© 2021. Published by The Company of Biologists under the terms of the Creative Commons Attribution License (https://creativecommons.org/licenses/by/4.0/).

o Please state if quantifications were performed blinded.

Authors' response:
Quantifications were indeed performed blinded. We apologize for not mentioning that. We have included a statement in the methods section.

Discussion section:

o Please consider the following study when addressing the outcome and impact of the results: Hortells et al., 2020. "A specialized population of Periostin-expressing cardiac fibroblasts contributes to postnatal cardiomyocyte maturation and innervation" PNAS, 117(35):21469-21479. doi:10.1073/pnas.2009119117.

Authors' response:
We have included the study in the discussion.

Second decision letter

MS ID#: DEVELOP/2020/198937

MS TITLE: Prrx1b restricts fibrosis and promotes Nrg1-dependent cardiomyocyte proliferation during zebrafish heart regeneration

AUTHORS: Dennis E.M. de Bakker, Mara Bouwman, Esther Dronkers, Filipa C. Simões, Paul R. Riley, Marie-José Goumans, Anke M. Smits, and Jeroen Bakkers
ARTICLE TYPE: Research Article

I am happy to tell you that your manuscript has been accepted for publication in Development, pending our standard ethics checks.

Reviewer 1

Advance summary and potential significance to field

This revision is a significant improvement upon the original submission. The authors now demonstrate co-expression of Prxx1b and Nrg1 which strengthens the main hypothesis (and title) of the paper. Knockout of Prxx1b impairs Nrg1 expression and heart regeneration. This is the first manuscript to uncover how Nrg1, a potent cardiac mitogen, may be regulated in zebrafish.

Comments for the author

There are no revisions necessary.

Reviewer 2

Advance summary and potential significance to field

The authors have addressed all comments satisfactorily and I recommend this manuscript for publication in current form.

Comments for the author

No more comments.

© 2021. Published by The Company of Biologists under the terms of the Creative Commons Attribution License (https://creativecommons.org/licenses/by/4.0/).

Reviewer 3

Advance summary and potential significance to field

The revised maniscript clarifies pending concerns and questiomns from the reviewers. Importantly it now adds more information on prrx1b expression (location and cell types) and adds control experiments for siRNA in vitro exprreriments. The Methods section is now also improved and contains important information regarding scar quantification.

Comments for the author

All concerns have been answered.

© 2021. Published by The Company of Biologists under the terms of the Creative Commons Attribution License (https://creativecommons.org/licenses/by/4.0/).
